# Recent Advances in Lignin-Based Wood Adhesives: Curing Behavior and Water Resistance

**DOI:** 10.3390/polym18212718

**Published:** 2026-11-02

**Authors:** Yan Ma, Qian Zhang, Zhichao Lou

**Affiliations:** 1 National-Provincial Joint Engineering Research Center of Biomaterials for Machinery Package, Nanjing Forestry University, Nanjing 210037, China; mayan@njfu.edu.cn; 2 Aica Nanjing Co., Ltd., Nanjing 210047, China; zhangqianlulu@126.com

**Keywords:** lignin, wood adhesives, curing behavior, crosslinked networks, water resistance

## Abstract

Lignin is a renewable aromatic feedstock for wood adhesives, but its structural heterogeneity complicates control over curing and water resistance. This review examines how lignin structure and functionalization govern curing reactions, kinetics, and crosslinked network development in lignin-based wood adhesives, and how these processes relate to bondline formation and wet bonding performance. Across condensation, epoxy, and polyurethane-based systems, molecular weight, aromatic condensation, and reactive-site accessibility influence lignin incorporation into polymer networks. However, chemical conversion, gelation, and effective network formation do not necessarily proceed in parallel; adhesive flow, penetration, and wood moisture further affect bondline development. Although crosslinking can reduce swelling and improve wet strength, faster curing or higher reactivity does not invariably yield better water resistance. The wet performance of wood joints also depends on network chemical stability, residual hydrophilic groups, interfacial bonding, and moisture-induced deformation and damage. Comparisons among studies remain limited by differences in lignin feedstocks, processing conditions, and water-exposure protocols. Future research should link curing kinetics, network architecture, moisture response, and interfacial failure under controlled testing conditions to establish verifiable structure–property relationships and guide formulation and processing.

## 1. Introduction

The curing behavior and wet bonding stability of wood adhesives directly influence the structural performance and long-term durability of engineered wood composites, including plywood, particleboard, and fiberboard [1]. The wood products industry continues to rely heavily on urea–formaldehyde (UF), phenol–formaldehyde (PF), and melamine–urea–formaldehyde (MUF) resins [1,2]. However, their dependence on petrochemical feedstocks and concerns over formaldehyde emissions from certain resin formulations have driven increasing interest in wood adhesives derived from renewable resources [3,4]. Among these renewable alternatives, lignin has attracted particular attention owing to its aromatic backbone and abundant oxygen-containing functional groups, which enable it to partially replace petroleum-derived phenol in phenolic resins or participate as a reactive component in polymer network formation [5,6]. Substituting lignin for phenol offers opportunities to reduce reliance on fossil resources, promote the value-added utilization of lignin, and potentially lower raw material costs, thereby further stimulating research into lignin-based wood adhesives [7,8,9]. However, its application remains constrained by structural heterogeneity and difficulties in controlling its chemical reactivity [10]. Cleavage of aryl ether linkages, condensation of aromatic structures, and functional group transformations during pulping and biorefinery processes result in substantial differences among technical lignins in molecular weight distribution, phenolic hydroxyl group composition, and the availability of reactive aromatic sites [11,12]. Studies on lignin fractionation have further demonstrated that molecular weight, the degree of aromatic condensation, and phenolic hydroxyl content do not exhibit simple linear relationships with adhesion behavior on resin surfaces [13]. Therefore, the reactive potential of lignin is governed not only by the abundance of functional groups but also by reactive-site accessibility, molecular structure, and the characteristics of the specific adhesive system.

In recent years, lignin-based wood adhesives have evolved from conventional lignin-modified phenol–formaldehyde (PF) resins to formaldehyde-free condensation systems, epoxy resins, polyurethanes, and other multicomponent reactive systems. Accordingly, network formation pathways have expanded beyond condensation at aromatic reactive sites to include epoxy ring-opening reactions, hydroxyl-mediated addition reactions, and crosslinking involving multiple functional groups [14]. Strategies for controlling lignin reactivity include chemical modifications such as phenolation, demethylation, hydroxymethylation, and epoxidation, as well as fractionation based on differences in solubility. While these approaches can improve the availability of reactive sites, they also alter lignin solubility, intermolecular interactions, and the conditions governing network formation [10]. Amine-cured epoxy resins prepared from different lignin fractions exhibit distinct thermomechanical properties, highlighting the influence of lignin molecular structure on the characteristics of the cured networks [15]. Furthermore, unlike systems that require prior functionalization and external curing agents, lignin with a low degree of condensation can undergo softening, flow, and further condensation during hot pressing, enabling direct wood bonding. This demonstrates that the structural evolution of lignin itself can contribute to the formation of the adhesive network [16]. The development of waterborne lignin-based epoxy resins and non-isocyanate polyurethane (NIPU) adhesives has further broadened the reaction pathways and processing approaches available for lignin-based adhesive systems [17,18]. Collectively, these studies indicate that lignin utilization is no longer limited to partially replacing components in conventional resins but is increasingly focused on optimizing bonding performance through functional group design, selection of curing reactions, and control of network structure.

As reactive systems have become increasingly diverse, research on lignin-based adhesives has expanded beyond final bonding performance to encompass curing kinetics, gelation, and the evolution of network structure. The incorporation of lignin can alter the rheological evolution of phenol–formaldehyde resins during curing. Furthermore, isoconversional kinetic analyses have revealed a pronounced dependence of the apparent activation energy on the degree of conversion in lignin-based novolac resins [19,20]. Pérez et al. [21] compared different novolac systems and found that the incorporation of lignin substantially altered the relationship between chemical conversion and the formation of a percolated network. Studies on PF resins modified with demethylated lignin have also demonstrated that changes in functional group structure can affect the apparent activation energy of curing. However, kinetic parameters do not directly reflect the effective crosslinking density of the resulting network [22,23]. During wood bonding, curing reactions are coupled with adhesive flow, wetting, and moisture migration within the wood. Consequently, lignin type, degree of modification, and substrate moisture content can all influence the thermal reaction behavior and processing characteristics of the resin [24,25]. Moreover, the spatial distribution of adhesives within wood pores is influenced by wood anatomical structure and adhesive properties, while adhesive penetration and network formation compete on the timescale of bondline development [26]. Therefore, elucidating the relationships among curing conversion, gelation behavior, effective network formation, and the structure of the resulting bondline is essential for understanding the curing characteristics of lignin-based adhesives.

Water resistance remains a major factor limiting the practical application of lignin-based wood adhesives and is a key consideration in adhesive structural design and performance optimization. In recent years, strategies such as multifunctional crosslinking, interpenetrating network formation, and compositional optimization have been employed to improve the moisture stability and wet bonding performance of lignin-based adhesives. Some systems have demonstrated promising resistance to hydrothermal exposure [27]. However, the moisture stability of cured networks does not necessarily correspond to the load-bearing capacity of wood joints under wet conditions. Moreover, differences in adhesive performance following water immersion and hydrothermal treatment are also influenced by bondline structure and testing conditions [28,29]. Previous studies on wood bonding have shown that moisture not only alters the viscoelastic response of cured polymers but also induces hygroscopic deformation of wood and changes in local interfacial stresses. These effects highlight the scale-dependent differences between the intrinsic properties of the cured adhesive layer and the macroscopic bonding performance of wood joints [30,31,32]. Recent studies on lignin-based adhesives have begun to investigate chemical linkages between lignin and wood cell walls and their influence on wet bonding performance [33]. Collectively, these studies indicate that the moisture stability of cured networks, the local mechanical response of wood–adhesive interfaces, and the load-bearing capacity of wood joints under wet conditions are not directly equivalent. Therefore, a comprehensive understanding of the water resistance of lignin-based wood adhesives requires consideration of the crosslinked network structure, interfacial bonding characteristics, and failure behavior under moisture exposure [34].

Previous reviews have established a broad foundation for lignin-based adhesive development. The review by Dangwilailux et al. [2] examines polymer architecture, interfacial interactions, and sustainability across several classes of bio-based wood adhesives, whereas Paez and Fatehi [35] summarize lignin modification and incorporation into different adhesive systems. Building on these contributions, the present review focuses specifically on the relationships between curing behavior and water resistance in lignin-based wood adhesives. Its contribution is to connect lignin reactivity, curing kinetics, network formation, and wet bonding performance, while distinguishing chemical conversion from effective network development and cured-resin properties from wood-joint performance. This review addresses three key questions: (i) through which chemical reactions lignin participates in network formation; (ii) how chemical conversion relates to the development of crosslinked networks; and (iii) how the structure and response of cured adhesive materials influence the wet bonding performance of wood joints. By addressing these questions, this review aims to provide a basis for the rational formulation and optimization of curing processes for lignin-based wood adhesives.

## 2. Molecular Structural Characteristics of Lignin and Their Influence on Curing Reactivity

The molecular structure and chemical reactivity of lignin are key factors influencing its curing behavior in wood adhesive systems. Lignins obtained from different sources and processing conditions exhibit substantial variations in functional group composition, aromatic structure, and molecular weight. These characteristics not only influence their potential reactivity but may also affect the processing characteristics of the resulting adhesives.

### 2.1. Structural Heterogeneity and Reactivity of Technical Lignins

**Figure 1:**
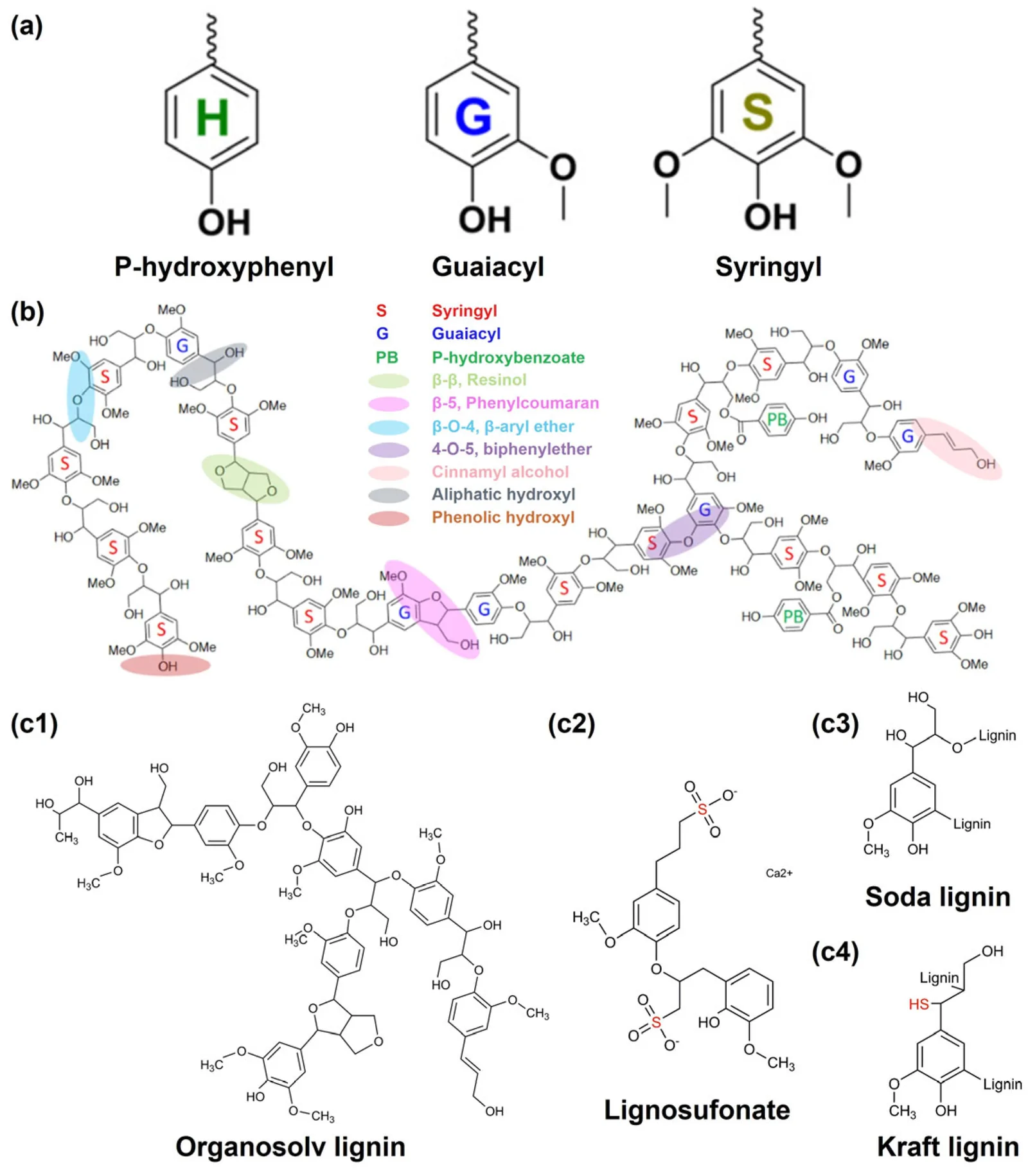
Structural features of lignin and representative technical lignins. (**a**) The three principal aromatic units: p-hydroxyphenyl (H), guaiacyl (G), and syringyl (S). Reprinted from Ref. [38]. (**b**) A representative hardwood lignin structure showing interunit linkages and the distribution of aliphatic and phenolic hydroxyl groups. Reprinted from Ref. [37]. (**c1**–**c4**) Simplified representative structures of technical lignins: (**c1**) organosolv lignin, (**c2**) calcium lignosulfonate, (**c3**) soda lignin, and (**c4**) kraft lignin. Reprinted from Ref. [39].

The curing reactivity of technical lignins is closely associated with the substitution patterns of their aromatic structures and the nature of their interunit linkages. Cleavage of aryl ether linkages, aromatic ring condensation, and functional group transformations during pulping and biorefinery processes result in substantial differences in the reactive sites retained by different technical lignins [11,12]. As shown in Figure 1a, lignin is primarily composed of p-hydroxyphenyl (H), guaiacyl (G), and syringyl (S) structural units, which are interconnected through aryl ether linkages, such as β-O-4, and carbon–carbon linkages, including β–5, β–β, and 5–5, to form a complex molecular structure (Figure 1b) [36,37]. The aromatic reactive positions differ among the three principal lignin units. The C3 and C5 positions of p-hydroxyphenyl (H) units and the C5 position of guaiacyl (G) units are potentially available for electrophilic aromatic substitution reactions, including certain aldehyde-mediated condensation reactions. In contrast, the C3 and C5 positions of syringyl (S) units are occupied by methoxy groups, limiting their availability for these reactions. However, the actual availability of these positions also depends on whether they are occupied by pre-existing interunit linkages [38]. In addition, aromatic carbon–carbon linkages formed during pulping may further reduce the number of available aromatic reactive sites [12]. These structural features affect both the number and accessibility of reactive sites. Occupation of an aromatic position by a methoxy substituent or a carbon–carbon linkage reduces the positions available for condensation, whereas steric hindrance restricts the approach of a reaction partner to a site that remains chemically available. These effects should therefore be distinguished when interpreting lignin reactivity.

In addition to aromatic reactive sites, the chemical reactivity of different types of hydroxyl groups is an important factor governing lignin participation in hydroxyl-mediated curing reactions, such as those involving isocyanates. Quantitative ^31^P NMR characterization revealed substantial differences in aliphatic hydroxyl, phenolic hydroxyl, and carboxyl group contents among six technical lignins [40]. Even among organosolv fractions with similar weight-average molecular weights, relatively small changes in total hydroxyl content concealed marked differences in the proportions of aliphatic and aromatic hydroxyl groups [41]. These compositional differences matter because hydroxyl groups are not equally reactive or accessible. During the direct reaction of two kraft lignins with excess 4,4′-diphenylmethane diisocyanate (MDI), aliphatic hydroxyl groups were completely consumed within 30 min, whereas residual phenolic hydroxyl groups remained after 90 min [42]. Alongside differences in intrinsic chemical reactivity, neighboring substituents can restrict access to phenolic hydroxyl groups: syringyl, guaiacyl, and p-hydroxyphenyl units contain two, one, and no adjacent methoxy groups, respectively. The lower reactivity of syringyl phenolic hydroxyl groups in the investigated MDI reactions was associated with stronger steric shielding. As reaction proceeds, the developing structures may further hinder access to remaining hydroxyl groups, while increasing viscosity separately restricts molecular diffusion. Thus, hydroxyl content and type provide a starting point for assessing lignin reactivity, but their actual utilization also depends on local steric accessibility and the evolving reaction environment.

Differences among technical lignins involve both molecular structure and the presence of non-lignin constituents [43]. Figure 1(c1–c4) shows simplified representative structures of organosolv lignin, calcium lignosulfonate, soda lignin, and kraft lignin, respectively, illustrating processing-related differences in interunit linkages and functional groups [39]. Beyond these structural differences, a comparative analysis of 13 technical lignins revealed substantial variations in the mass fractions of acid-insoluble lignin, acid-soluble lignin, carbohydrates, moisture, and ash [44]. Lignin purity should be reported on a clearly stated dry or wet basis, together with the types and amounts of non-lignin constituents. Comparable purity, however, does not imply equivalent reactivity, because lignins can differ in molecular-weight distribution, hydroxyl-group composition, and reactive-site accessibility. Composition and structural characterization should therefore be considered together when evaluating technical lignins for adhesive preparation.

Fractionation studies have further revealed relationships between lignin molecular structure, surface interactions, and the properties of cured materials. Wibowo and Park [13] reported that the acetone-soluble lignin fraction exhibited a lower molecular weight, higher phenolic hydroxyl content, and a greater degree of aromatic condensation. This fraction also showed higher adhesion forces and work of adhesion on model phenol–formaldehyde (PF) and urea–formaldehyde (UF) resin surfaces. Regarding the properties of cured materials, Lang et al. [45] found that polyurethanes prepared from hardwood lignosulfonates with higher hydroxyl contents exhibited lower storage moduli. Based on atomic force microscopy (AFM) and scanning electron microscopy (SEM) observations, the authors associated this behavior with lignin aggregation and dispersion characteristics. These two studies, focusing on initial surface interactions and the response of cured materials, respectively, highlight the combined influence of molecular structure and component dispersion on material behavior. Therefore, evaluating the curing reactivity of technical lignins requires consideration of aromatic reactive-site distribution, hydroxyl group reaction selectivity, and the solubility and dispersion characteristics of lignin components.

### 2.2. Modification and Utilization of Reactive Sites in Lignin

Owing to differences in aromatic reactive sites, functional group accessibility, and solubility among technical lignins, chemical modification is commonly employed to improve their reactivity and processability. Representative modification and fractionation strategies, together with their applications and main findings, are summarized in Table 1. Common chemical modification strategies include phenolation and demethylation. Phenolation can introduce additional phenolic structures while altering pre-existing interunit linkages, whereas demethylation converts methoxy groups into phenolic hydroxyl groups. In the acid-catalyzed phenolation of biorefinery wheat straw lignin, Zhang et al. [46] identified phenol incorporation into side-chain and aromatic structures, accompanied by changes in pre-existing interunit linkages. The influence of lignin fraction characteristics was demonstrated by Wang et al. [47]: lower-molecular-weight, less-condensed fractions were more readily incorporated into LPF resins after phenolation. For kraft lignin, sulfobutylation pretreatment followed by demethylation combined improved water solubility with functional-group conversion for PF resin preparation [48]. These studies show that changes in phenolic functionality should be considered together with lignin structure and solubility.

Hydroxymethylation and reactions between lignin and glyoxal modify the substitution patterns of aromatic reactive sites, providing distinct pathways for subsequent condensation and chemical bond formation. Paananen et al. [49] confirmed the hydroxymethylation of lignin aromatic structures during co-hydroxymethylation with phenol. Under the investigated conditions, however, methylene linkages formed predominantly between hydroxymethylated phenol units. For lignin–glyoxal systems, the model-compound and resin investigations of Siahkamari et al. [50] provided NMR evidence for glyoxal-derived linkages, including those in the cured resin. These findings distinguish functional-group introduction from subsequent intermolecular bond formation, indicating that modification should be evaluated through the linkages formed during resin preparation and curing.

In non-phenolic resin systems, strategies for regulating lignin reactivity primarily focus on the direct utilization of lignin hydroxyl groups in crosslinking reactions or their conversion into specific reactive functionalities. Direct hydroxyl participation was supported by spectroscopic analyses of the reaction between acetone-fractionated hardwood kraft lignins and epichlorohydrin in the study of Watumlawar and Park [51], with new oxygen-containing linkages detected after reaction. Hydroxypropylation provides a contrasting route through hydroxyl derivatization: the comparison of unmodified and modified kraft lignins by Gouveia et al. [52] showed that treatment conditions affected the thermomechanical properties and wood lap-shear strength of polyurethane adhesives. These studies demonstrate that lignin hydroxyl groups can participate directly in crosslinking or be modified to alter their accessibility and compatibility with the target resin system.

Amination enables lignin to participate in different adhesive systems, but its preparation route and subsequent role vary among studies. Chen et al. [53] prepared aminated enzymatic hydrolysis lignin through Fenton oxidation followed by reductive amination with ammonia and sodium borohydride. The resulting lignin amine was reacted with epoxidized soybean oil (ESO) to obtain an EHL–ESO component for soy protein isolate (SPI) adhesives. Li et al. [54] and Aladejana et al. [55] incorporated aminated lignin into NIPU systems. By comparison, the ENR adhesive system reported by Xu et al. [56] used KH550-functionalized lignin to introduce amino functionality for crosslinking epoxidized natural rubber. These routes therefore differ in how amino functionalities are introduced and in the reactions through which lignin becomes incorporated into the adhesive network. The corresponding preparation strategies and outcomes are compared in Table 1.

In addition to chemical modification, solvent fractionation can selectively enrich lignin fractions with different molecular weights and functional group compositions, providing feedstocks with more narrowly distributed structural characteristics for subsequent reactions without directly introducing new reactive groups [47]. The fractionation–phenolation results discussed above link the characteristics of the selected fractions to their subsequent incorporation into LPF resins. A different combination, fractionation followed by epoxidation, enabled an ethanol-soluble fraction of enzymatic hydrolysis lignin to participate in an amine-terminated NIPU system in the study of Zhang et al. [57]. These combinations illustrate how feedstock selection and subsequent functionalization can be coordinated to prepare reactive lignin components for different resin systems.

## 3. Curing Reactions and Structural Development of Lignin-Based Wood Adhesives

The curing of lignin-based wood adhesives involves the transformation of reactive functional groups, the formation of intermolecular linkages, and the development of crosslinked networks [58]. These processes are jointly influenced by lignin molecular structure, curing reaction pathways, and wood bonding conditions [59,60]. During wood bonding, curing is also accompanied by adhesive wetting, penetration into wood pores, and moisture migration. Consequently, both the chemical structure and spatial distribution of the adhesive within the bondline contribute to wood joint formation.

### 3.1. Curing Reaction Pathways and Chemical Selectivity of Lignin-Based Adhesives

The participation of lignin in resin curing involves the formation of linkages within prepolymers and subsequent crosslinking reactions initiated by curing agents. Bansode et al. [61] characterized the prepolymer structure using Fourier-transform infrared (FTIR) and nuclear magnetic resonance (NMR) spectroscopy, confirming the formation of methylene linkages. They further investigated the subsequent curing behavior using differential scanning calorimetry (DSC). Previous studies have also demonstrated that lignin incorporation can alter prepolymer composition and subsequent curing behavior [61,62,63]. Tejado et al. [64] compared the curing behavior of novolac-type lignin–phenol–formaldehyde (LPF) resins using hexamethylenetetramine (HMTA) and 4,4′-diphenylmethane diisocyanate (MDI) as curing agents. They found that the MDI-cured system exhibited faster curing at lower temperatures. MDI can react with hydroxyl groups in lignin to form urethane linkages and further react with hydroxyl groups in LPF resins to construct crosslinked structures. These two curing pathways involve network formation through further crosslinking of phenolic resin structures and isocyanate–hydroxyl reactions, respectively, highlighting how the choice of curing agent influences the utilization of reactive sites in lignin.

**Figure 2:**
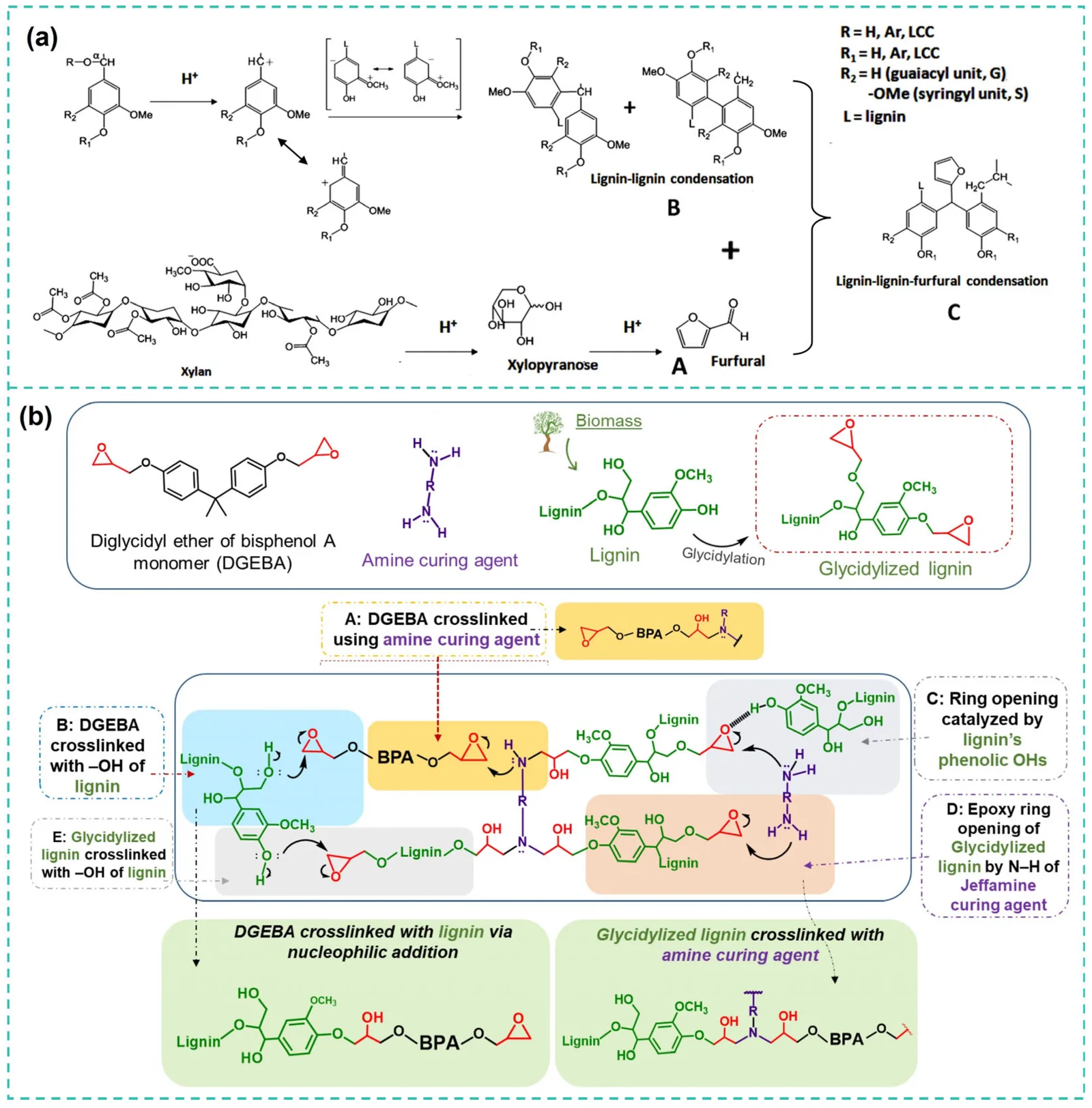
Representative condensation and epoxy crosslinking pathways in lignin-based adhesive systems. (**a**) Proposed condensation pathways in the lignin–furfural system under acidic conditions, including lignin–lignin self-condensation and furfural-mediated co-condensation. Reprinted from Ref. [66]. (**b**) Proposed crosslinking pathways involving lignin, glycidylated lignin, DGEBA, and diamine. Reprinted from Ref. [68].

The choice of aldehyde and reaction conditions can influence lignin condensation pathways and the resulting chemical linkages [65,66]. Dongre et al. [66] prepared resins from hot-water-extracted sugar maple lignin under varying acidity and furfural addition conditions. They used size-exclusion chromatography (SEC) and mechanical testing of resin-reinforced glass fiber specimens to investigate structural characteristics and curing-related performance. The authors proposed that lignin self-condensation, furfural-mediated co-condensation, and changes in pre-existing linkages collectively contributed to resin formation under acidic conditions (Figure 2a). Ghahri and Park [67] glyoxalated acetone-fractionated hardwood kraft lignin. Fourier-transform infrared (FTIR) spectroscopy, X-ray photoelectron spectroscopy (XPS), and solid-state ^13^C NMR analyses provided evidence for the formation of ether linkages between glyoxalated lignin molecules. Among the investigated fractions, adhesives prepared from the acetone-insoluble fraction exhibited the highest plywood bonding strength. These two studies illustrate the characteristics of acid-catalyzed structural rearrangement and the formation of oxygen-containing linkages following glyoxalation, respectively, highlighting the need to match aldehyde selection with lignin fraction structure and reaction conditions [15,67].

**Figure 3:**
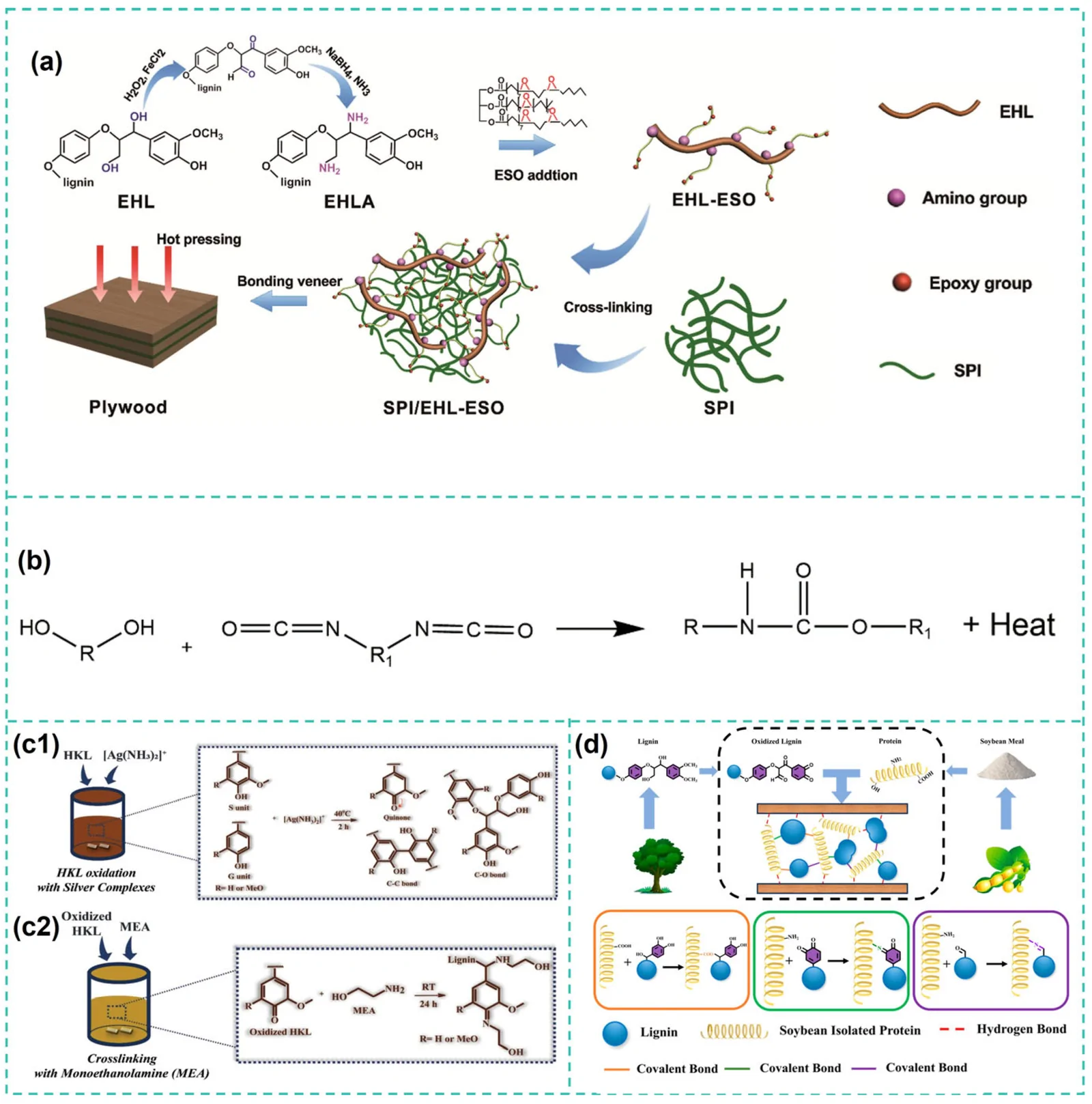
Representative preparation and crosslinking pathways of lignin-based protein and polyurethane adhesive systems. (**a**) Preparation of an enzymatic hydrolysis lignin–epoxidized soybean oil (EHL–ESO) component through lignin amination and subsequent reaction with epoxidized soybean oil, followed by crosslinking with soy protein isolate (SPI). Reprinted from Ref. [53]. (**b**) Hydroxyl–isocyanate addition forming a urethane linkage. Reprinted from Ref. [37]. (**c1**,**c2**) Proposed oxidative activation and subsequent crosslinking reactions of hardwood kraft lignin. Reprinted from Ref. [69]: (**c1**) oxidation of lignin by silver–ammonia complexes, promoting the formation of quinone structures and intermolecular linkages; (**c2**) reaction between oxidized lignin and monoethanolamine (MEA). (**d**) Preparation and proposed crosslinking mechanism of soybean meal–oxidized lignin adhesives, illustrating covalent linkages and hydrogen-bonding interactions between oxidized lignin and protein components. Reprinted from Ref. [70].

Epoxy-based approaches introduce functional groups capable of undergoing ring-opening reactions, enabling lignin to be incorporated into amine-cured or multicomponent crosslinked networks. The proposed pathways in Figure 2b distinguish the roles of unmodified and glycidylated lignin in epoxy network formation. They include epoxy–amine addition involving glycidylated lignin, hydroxyl-mediated epoxy ring opening, and hydroxyl-assisted catalysis [68]. Gioia et al. [15] epoxidized fractionated lignin and subsequently cured the resulting epoxy derivatives with a polyether diamine. Nuclear magnetic resonance (NMR) spectroscopy, size-exclusion chromatography (SEC), and thermomechanical analysis were employed to investigate the relationships between lignin fraction structure and the properties of the resulting epoxy materials. In the EHL–ESO/SPI system summarized in Table 1, aminated lignin is used to prepare an epoxy-containing intermediate for subsequent protein crosslinking [53]. The proposed curing pathway involves residual epoxy groups reacting with protein functional groups (Figure 3a). In the former system, lignin-derived epoxy components serve as network-building units, whereas the latter employs a multifunctional intermediate to connect lignin and protein components, resulting in distinct connectivity between the constituent components.

In polyurethane systems, the chemical nature and accessibility of lignin hydroxyl groups influence the extent of their reaction with isocyanates. The underlying bond-forming reaction is the addition of a hydroxyl group to an isocyanate group to form a urethane linkage (Figure 3b) [37]. In lignin-containing formulations, this reaction provides a route for incorporating accessible lignin hydroxyl groups into the polyurethane network. Vieira et al. [71] reacted oxyalkylated kraft lignin polyols with polymeric methylene diphenyl diisocyanate (pMDI) and demonstrated that varying the NCO/OH ratio altered the gelation behavior and properties of the cured materials. Residual 1,8-diazabicyclo[5.4.0]undec-7-ene (DBU) catalyst in the crude polyols also contributed to an increase in the reaction rate. In contrast, non-isocyanate routes alter the reactive role of lignin. In the NIPU system of Li et al. [54] summarized in Table 1, introduced amino groups react with cyclic carbonate groups to form hydroxyurethane linkages.

Oxidative treatments can promote intermolecular linkage formation in lignin through radical coupling or the generation of new reactive oxygen-containing structures. In a pilot-scale study involving laccase-treated beech fibers, Felby et al. [72] observed enhanced lignin polymerization during treatment and hot pressing, as indicated by changes in the molecular weight of extractable lignin. The authors associated fiber bonding with coupling reactions involving phenoxy radicals. Watumlawar et al. [69] oxidized the acetone-soluble fraction of hardwood kraft lignin using a silver–ammonia complex. Based on Fourier-transform infrared (FTIR) spectroscopy, ^13^C NMR spectroscopy, and gel permeation chromatography (GPC) analyses, they proposed reaction pathways involving the formation of quinone structures and intermolecular linkages. Monoethanolamine (MEA) was subsequently introduced to prepare adhesives (Figure 3(c1,c2)). These two approaches utilize enzyme-catalyzed oxidative polymerization and the modification of reactive sites following oxidation, respectively, enabling lignin-derived reactive structures to be incorporated into adhesive components.

When oxidized lignin is combined with proteins, changes in oxygen-containing functional groups can facilitate covalent bond formation and intermolecular association. Zhang et al. [70] oxidized sodium lignosulfonate using hydrogen peroxide and characterized the resulting products through molecular weight and spectroscopic analyses. Changes in the N 1s and O 1s spectral components were observed in adhesive layers formed from oxidized lignosulfonate and soybean meal. Based on these observations, the authors proposed a bondline formation mechanism involving reactions between aldehyde- or quinone-containing structures and amino groups in the proteins, together with esterification and hydrogen bonding (Figure 3d). In contrast to the EHL–ESO system, in which epoxy groups play a major role in protein crosslinking, oxidized lignin systems create reactive sites through changes in the composition of existing oxygen-containing functional groups. These differences illustrate distinct chemical linkage pathways in lignin–protein composite adhesives.

### 3.2. Curing Kinetics and Structural Evolution of Crosslinked Networks

Lignin structure, functionalization, and formulation composition influence curing kinetics, with effects that vary across phenolic and epoxy systems and with the stage of conversion (Table 2). In phenolic systems, comparisons of lignin substitution levels and demethylation treatments linked kinetic changes to reactive-group composition and viscosity evolution [20,22]. Demethylation reduced the apparent activation energy relative to the unmodified LPF resin and shortened gelation time [22]. In epoxy systems, both lignin source and curing-agent structure affected the conversion dependence of activation energy [73]. The effect of lignin-based epoxy loading was also stage-dependent: a content of 25 wt% accelerated early-stage curing, whereas contents above 50 wt% inhibited later-stage reactions [74]. Thus, lignin addition does not exert a uniformly accelerating or inhibiting effect; its influence depends on formulation composition and curing progress.

Kinetic models can also be used to establish relationships between reaction progress and curing temperature profiles. Alonso et al. [75] analyzed resol-type phenol–formaldehyde (PF) and lignin–phenol–formaldehyde (LPF) resins using the Ozawa, Kissinger–Akahira–Sunose (KAS), and Friedman methods to determine the relationship between activation energy and the degree of cure. They further employed thermomechanical analysis (TMA) and differential scanning calorimetry (DSC) to relate gelation behavior to chemical conversion. Huo et al. [76] investigated the curing of a lignin–cardanol-based novolac epoxy resin with methyl tetrahydrophthalic anhydride. They determined kinetic parameters using the Friedman method and Málek analysis and described the curing reaction using the Šesták–Berggren model. The kinetic equation derived from non-isothermal data was subsequently applied to predict isothermal conversion profiles, which showed good agreement with the experimental curves. These two studies demonstrate how thermal analysis results can be used to characterize the curing progress of specific formulations through conversion-dependent kinetic analysis and validation across different temperature programs, respectively.

**Figure 4:**
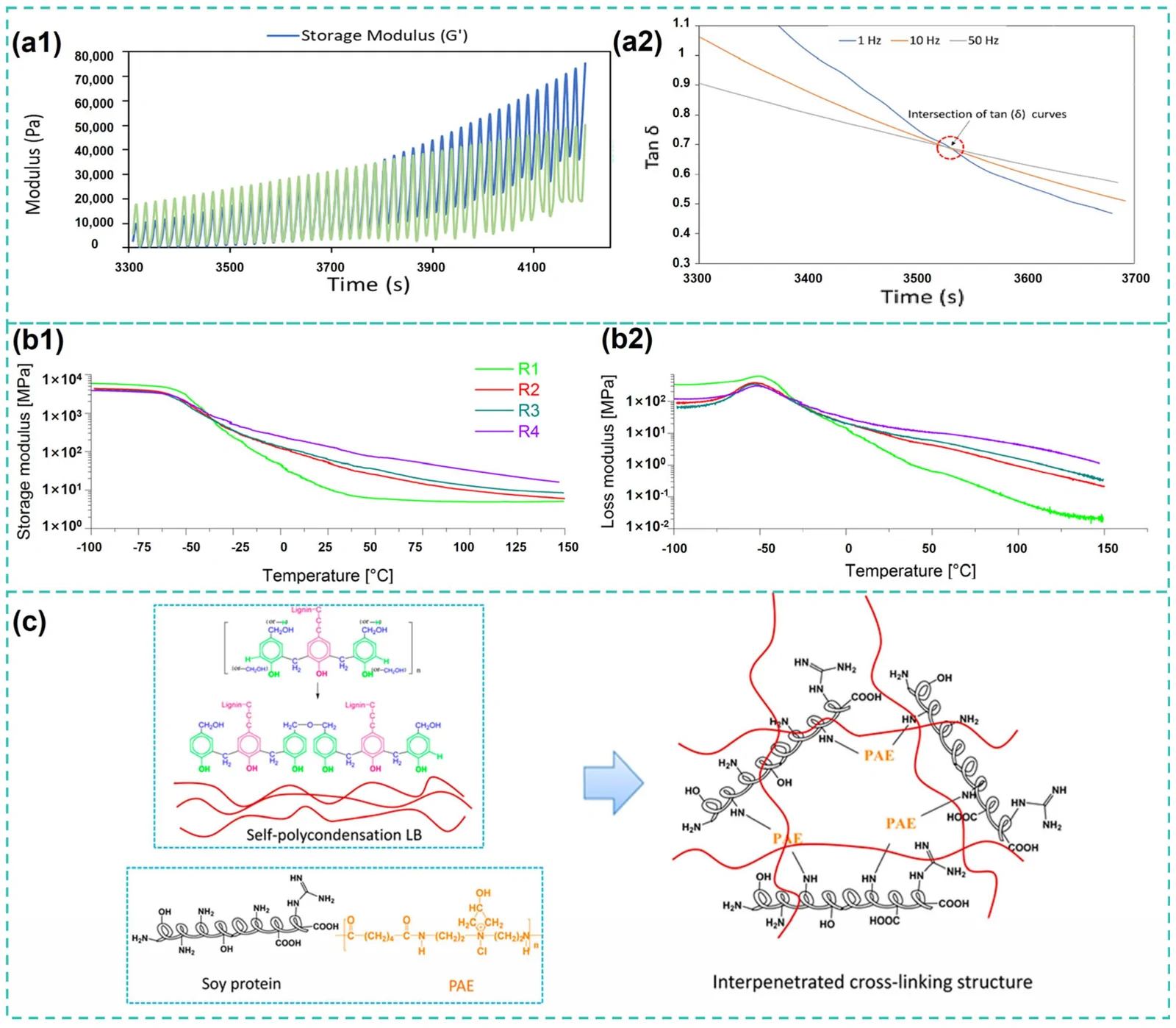
Gelation, thermomechanical responses of cured materials, and multicomponent network construction in lignin-based thermosetting systems. (**a1**,**a2**) Gel-point identification by multifrequency rheology in a lignin-based epoxy system. Reprinted from Ref. [78]: (**a1**) evolution of the storage modulus G′ during curing of a hardwood lignin-based epoxy formulation measured at 1, 10, and 50 Hz; (**a2**) loss factor tan δ curves at different frequencies, with their intersection indicating the gel point. (**b1**,**b2**) Dynamic mechanical temperature spectra of epoxy resins prepared from different solvent-fractionated lignins. Reprinted from Ref. [15]: (**b1**) storage modulus and (**b2**) loss modulus; R1–R4 denote resins prepared from the different lignin fractions. (**c**) Proposed interpenetrating network structure comprising a self-condensed lignin-based resin (LB) network and a polyamidoamine–epichlorohydrin (PAE)-crosslinked soy protein network. Reprinted from Ref. [79].

Gelation represents the transition from intermolecular linkage formation to the development of a percolated network, with the corresponding degree of conversion depending on the functionality of the reactive components and their modes of connectivity. Pérez et al. [21] combined thermomechanical analysis (TMA) with residual curing enthalpy measurements to determine the gel-point conversions of PF, LN, and MLN resins containing 9 wt% hexamethylenetetramine (HMTA). The corresponding values were 51.3%, 30.3%, and 30.2%, respectively, indicating that lignin incorporation altered the overall degree of conversion required for network percolation. In rheological characterization, the Winter–Chambon criterion identifies the gel point based on the frequency dependence of viscoelastic moduli at the critical gel state. Nasseri et al. [77] applied multifrequency rheological measurements to determine the gel points of formaldehyde-based wood adhesives. Nikafshar et al. [78] further investigated the curing behavior of lignin-based epoxy systems using multifrequency rheological measurements. They monitored the evolution of the storage modulus with time for a hardwood lignin-based epoxy formulation at 1, 10, and 50 Hz (Figure 4(a1)) and identified the gel point from the intersection of tan δ curves measured at different frequencies (Figure 4(a2)). By relating gel-point identification to changes in the frequency dependence of the loss factor, this study provided rheological evidence for the transition of lignin-based epoxy systems from a viscous fluid to a percolated network.

Vitrification alters network development after gelation by restricting polymer chain segmental mobility, making the relationship between the curing temperature and the glass transition temperature (Tg) of the material an important factor governing later-stage curing reactions. Alonso et al. [80] combined thermomechanical analysis (TMA) and differential scanning calorimetry (DSC) to construct time–temperature–transformation (TTT) diagrams for resol-type phenol–formaldehyde (PF) and lignin–phenol–formaldehyde (LPF) resins. These diagrams incorporated gelation lines, vitrification boundaries, and isoconversion lines within a common time–temperature framework. These results distinguish network percolation from vitrification as separate transitions and demonstrate that curing temperature and time jointly govern the evolution of the material from continued network growth toward a state of restricted molecular mobility.

Epoxidation of lignin can alter the early-stage curing rate of blended epoxy systems, whereas differences in molecular weight and structure among lignin fractions influence the thermomechanical response of the cured materials. Gouveia et al. [81] employed temperature-modulated optical refractometry to monitor the early-stage curing of epoxy adhesives blended with unmodified or epoxidized kraft lignin at 25 °C. They found that unmodified lignin reduced the curing rate, whereas epoxidation improved the curing behavior. Gioia et al. [15] observed differences in rubbery plateau moduli and loss modulus peak temperatures among epoxy materials prepared from different lignin fractions (Figure 4(b1,b2)). They associated the thermomechanical behavior at elevated temperatures with the molecular weight and structural rigidity of the lignin fractions. Together, these studies indicate, from the perspectives of curing behavior and the response of cured materials, respectively, that lignin functionalization and macromolecular structure jointly influence network formation and segmental relaxation behavior.

In multicomponent systems, the structure of the cured network is governed by interactions and linkages among different reactive components, as well as their spatial organization. Zhang et al. [79] modified soy flour adhesives using a lignin-based phenolic resin and polyamidoamine–epichlorohydrin (PAE). Based on Fourier-transform infrared (FTIR) spectroscopy, thermogravimetric analysis (TGA), and X-ray diffraction (XRD) results, they proposed a structural model in which self-condensed lignin-based resin structures interpenetrate a crosslinked protein network (Figure 4c). The authors attributed the improved adhesive performance to the combined contributions of protein crosslinking, resin condensation, and penetration into the bonding region. These findings suggest that the role of lignin in composite adhesives depends on how it interacts and forms linkages with other components, as well as the spatial organization of the reactive constituents within the bondline.

### 3.3. Hot-Press Curing and the Development of Wood–Adhesive Interfacial Structures

**Figure 5:**
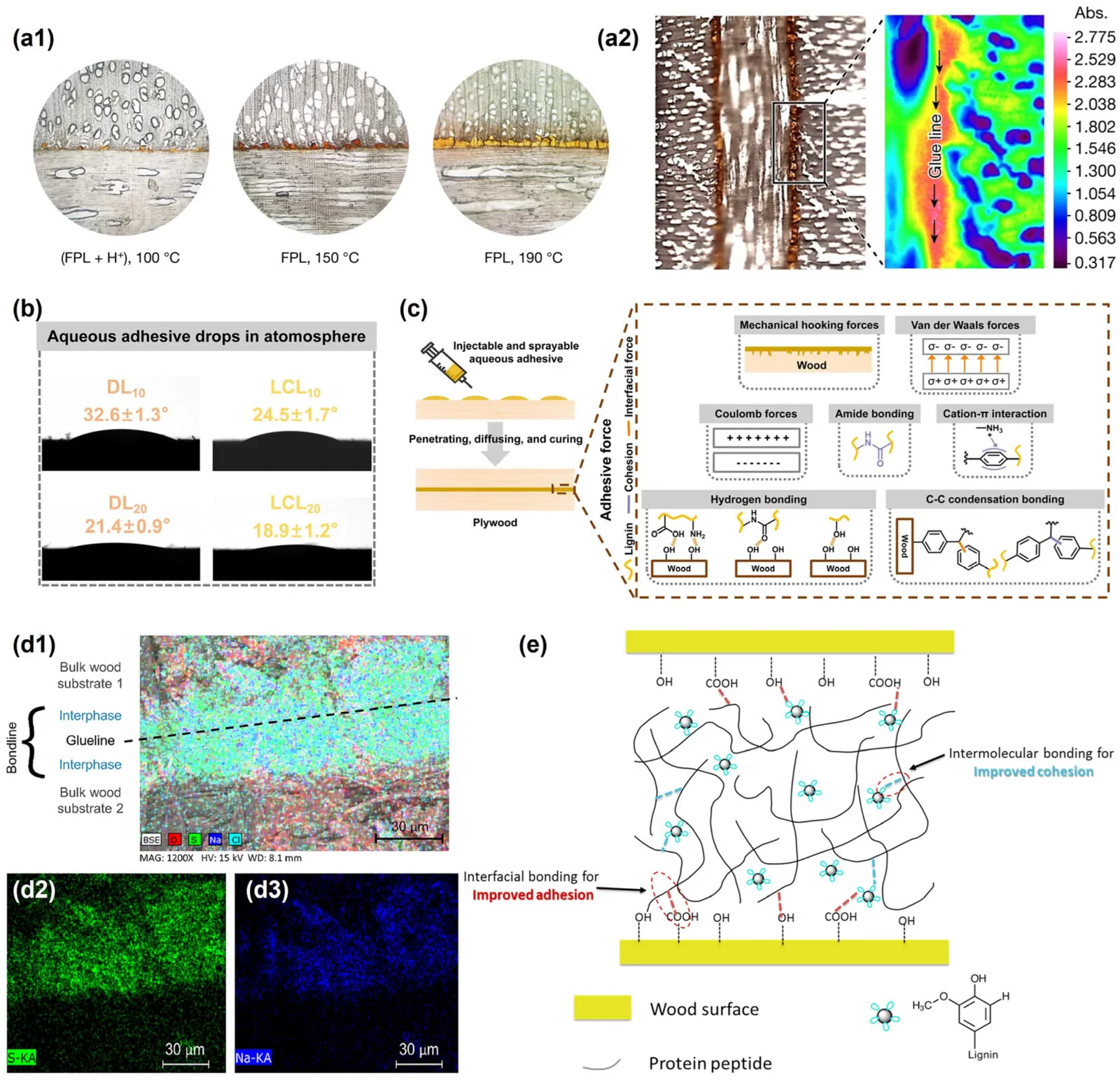
Bondline morphology, wetting behavior, and proposed bonding mechanisms of lignin-based wood adhesives. (**a1**) Optical micrographs of bondlines formed using formaldehyde-protected lignin (FPL) at different hot-pressing temperatures: acid-catalyzed FPL at 100 °C and FPL without added acid at 150 and 190 °C; (**a2**) scanning electron microscopy and Fourier-transform infrared microscopy images of the bondline region formed at 190 °C, with infrared mapping recorded at 1597 cm^−1^. Reprinted from Ref. [16]. (**b**) Contact angles of aqueous dioxane-extracted lignin (DL) and cysteine-functionalized lignin (LCL) adhesives on wood veneer; subscripts 10 and 20 denote adhesive concentrations of 10 and 20 wt%, respectively. Reprinted from Ref. [82]. (**c**) Proposed bonding mechanism of the aqueous LCL adhesive, illustrating penetration and curing together with physical and chemical interactions contributing to interfacial adhesion and adhesive cohesion. Reprinted from Ref. [82]. (**d1**) Bondline morphology with superimposed energy-dispersive X-ray spectroscopy elemental maps for a sodium lignosulfonate/softwood kraft lignin adhesive crosslinked with glycerol diglycidyl ether; (**d2**,**d3**) corresponding sulfur and sodium distributions, respectively. Reprinted from Ref. [83]. (**e**) Schematic illustration of intermolecular interactions within a camelina protein–lignin adhesive and interfacial interactions with wood surfaces, distinguishing contributions to adhesive cohesion and interfacial adhesion. Reprinted from Ref. [84].

During hot pressing, lignin softening and subsequent condensation jointly influence adhesive distribution and the fixation of the bondline structure. Yang et al. [16] directly bonded wood using an aqueous suspension of lignin with a low degree of condensation. They observed increased filling of wood vessels by softened lignin as the hot-pressing temperature increased from 100 to 190 °C. Hydrogenolysis and two-dimensional heteronuclear single-quantum coherence nuclear magnetic resonance (2D HSQC NMR) analyses before and after hot pressing provided evidence for an increased degree of condensation and the formation of additional carbon–carbon linkages (Figure 5(a1,a2)). Based on these findings, the authors proposed a bonding mechanism involving water-assisted softening, pore filling by softened lignin, and thermally induced crosslinking, thereby linking initial contact and spatial distribution to bondline curing. Structural development in this system depends on the mobility and reactive potential retained by lignin before hot pressing, thereby linking the initial degree of lignin condensation directly to bondline formation.

The dispersion structure and room-temperature reaction rate of waterborne adhesives jointly influence their flowability and pot life during application. Huo et al. [17] prepared waterborne lignin-based epoxy resins through emulsification, obtaining particle sizes of 321–696 nm and viscosities of 110–470 mPa·s. The length of the polyethylene glycol (PEG) segments in the emulsifier also influenced the thermomechanical properties of the cured materials. Ferruti et al. [83] used sodium lignosulfonate to disperse softwood kraft lignin and blended the resulting dispersion with glycerol diglycidyl ether. Using a doubling of the initial viscosity as the criterion for determining pot life, they found that increasing the proportion of sodium lignosulfonate extended the pot life from less than 3 min to 17–150 min. Two contributions were proposed for this extension: a lower concentration of reactive phenolic groups and physical shielding of kraft lignin reaction sites by the larger, branched lignosulfonate macromolecules [83]. The latter represents a formulation-level steric constraint that can reduce reaction probability and extend the processing window. These two formulation strategies regulate adhesive behavior during application through emulsion structure and reactive-component proportions, respectively, indicating that dispersion design and control of room-temperature curing jointly influence the conditions for adhesive contact with wood and subsequent redistribution.

Wetting and thermally induced bond formation constitute interconnected processes in the bonding behavior of functionalized lignin adhesives. Zhou et al. [82] prepared a waterborne adhesive containing 20 wt% cysteine-functionalized lignin. Contact angle measurements indicated improved wetting of the wood surface (Figure 5b), while molecular simulations further suggested enhanced hydrogen-bonding, electrostatic, and van der Waals interactions between lignin and wood constituents. The authors interpreted Fourier-transform infrared (FTIR) spectroscopy, X-ray photoelectron spectroscopy (XPS), and other structural analyses performed after hot pressing as evidence for the formation of amide-containing linkages and condensed structures. On this basis, they proposed a bonding mechanism in which polar functional groups facilitate initial interfacial contact, while thermally induced bond formation enhances bondline cohesion (Figure 5c). In contrast to systems based on lignin with a low degree of condensation, where bondline formation primarily relies on thermal softening and subsequent condensation, cysteine functionalization modifies both interfacial interactions and the linkage structures formed during curing, providing distinct structural explanations for improved wetting and enhanced cohesion.

Wood moisture conditions also influence the formation of isocyanate-based bondlines by participating in chemical reactions and altering local phase morphology. He and Yan [25] used differential scanning calorimetry (DSC) to investigate mixtures of polymeric methylene diphenyl diisocyanate (pMDI) and wood with different moisture contents. They found that wood moisture affected the curing kinetics, indicating that moisture conditions constitute an important aspect of the reaction environment for isocyanate-based adhesives. Ren and Frazier [85] compared free-standing adhesive films with wood bondlines in model and commercial moisture-curing polyurethane systems. Atomic force microscopy (AFM) revealed changes in the size and distribution of dispersed phases, while dynamic mechanical analysis (DMA) identified corresponding changes in relaxation transitions. Infrared spectroscopic analysis further indicated an increase in hydrogen-bonded urea structures within the wood bondlines. These studies of conventional polyurethane systems establish links between wood moisture conditions, bondline reactions, and microphase morphology, providing a reference for investigating the curing of lignin-based isocyanate adhesives under realistic wood-substrate conditions.

The spatial distribution of adhesives resulting from penetration into wood pores influences mechanical interlocking and bondline continuity at the bonding interface. Paris and Kamke [26] used X-ray computed tomography (X-ray CT) to reconstruct different wood–adhesive systems and found that a weighted assessment incorporating both the area of adhesive distribution and penetration depth provided a more comprehensive characterization of the three-dimensional adhesive distribution within different bonding regions. In lignin-based systems, Ferruti et al. [83] used scanning electron microscopy coupled with energy-dispersive X-ray spectroscopy (SEM–EDS) to observe adhesive penetration into adjacent wood tissues, with elements such as sulfur (S) and sodium (Na) predominantly distributed in the bonding region (Figure 5(d1–d3)). They also applied a cationic starch pre-coating during plywood fabrication to regulate adhesive retention at the bonding interface. Together, these studies highlight, through three-dimensional distribution analysis and local morphological characterization, respectively, the importance of considering penetration depth, adhesive distribution, and interfacial adhesive retention when evaluating the spatial structure of wood bondlines.

Interactions and chemical bridging at the cell-wall scale further influence the bonding mechanisms through which loads are transferred from the adhesive bondline to the wood substrate. Laborie et al. [86] investigated the interactions between wood and phenol–formaldehyde (PF) resins of different molecular weights using dynamic mechanical analysis (DMA) and solid-state ^13^C nuclear magnetic resonance (NMR) spectroscopy. They observed changes in the relaxation behavior of wood polymers induced by low-molecular-weight PF resins and proposed that these resins exhibited nanoscale mixing with amorphous cell-wall polymers. In a lignin-based system, Tang et al. [33] employed trimethylolpropane triglycidyl ether to mediate etherification reactions between lignin and cellulose, thereby covalently anchoring lignin to wood fibers and constructing a bonding network linked to the composite structure of the cell wall. Protein–lignin composite systems further illustrate the complementary roles of cohesion and adhesion. Zhu et al. [84] proposed that depolymerized lignin contributes to both intermolecular bonding among camelina protein molecules and interactions with the wood surface (Figure 5e), allowing internal bondline cohesion and adhesion to the substrate to contribute jointly to the formation of the bonded joint.

Strength development in actual bonded joints depends on the combined effects of chemical reactions, bondline formation, and interactions with the wood substrate. Saražin et al. [87] compared organosolv lignin-based non-isocyanate polyurethane (NIPU), tannin–hexamethylenetetramine, and urea–formaldehyde adhesives using DSC and the Automated Bonding Evaluation System (ABES). The systems exhibited different strength-development rates and suitable pressing conditions, demonstrating the value of measuring bond strength development alongside thermal curing behavior when evaluating wood-bonding processes.

## 4. Water Resistance and Wet-State Failure of Lignin-Based Wood Adhesives

The water resistance of lignin-based wood adhesives is governed by the combined effects of cured network structure, wood–adhesive interfacial characteristics, and moisture exposure conditions. Moisture can affect not only the structural stability and mechanical response of cured adhesives but also the load-bearing capacity of bonded joints under wet conditions through moisture-induced wood swelling and changes in interfacial stresses.

### 4.1. Evaluation Metrics and Water Exposure Conditions for Water Resistance

The water resistance of wood products should be assessed in relation to their intended applications and actual service environments. For indoor furniture and decorative products, the primary concern is bond stability under specified humidity conditions. For wood-based composites, such as plywood and particleboard, dimensional changes in the panels as a whole must also be considered. Outdoor or load-bearing bonded wood structures, in contrast, place more stringent demands on strength retention and durability under repeated moisture exposure. Accordingly, testing methods and evaluation metrics should be selected to reflect the service conditions of each application, and adhesive suitability should not be judged solely on the basis of a single wet-strength measurement or water absorption value [88,89].

For indoor furniture and non-load-bearing bonded wood products, water resistance is primarily evaluated in terms of bond strength and its retention following specified conditioning or short-term water immersion. EN 204 classifies thermoplastic wood adhesives for non-structural applications into four durability classes (D1–D4) based on bond strength measured under specified conditioning and water-exposure conditions [90]. Arif et al. [91] combined poly(itaconic acid)-functionalized kraft lignin with chitosan. The resulting wood-bonded joints exhibited a wet bond strength of approximately 1.70 ± 0.70 MPa after 6 h of water immersion, which increased to 2.39 ± 0.45 MPa after 7 days of redrying. The difference between these testing conditions highlights the distinct performance requirements associated with loading immediately after water immersion and loading after drying recovery. Strength measured after redrying therefore cannot be used as a direct substitute for load-bearing capacity under wet conditions.

Plywood bonding quality is evaluated using shear strength and wood failure percentage after specified water treatments, as addressed by ISO 12466-1 and ISO 12466-2 [92,93]. The oxidized sucrose–lignosulfonate system illustrates that strength after hot-water treatment can exceed the dry-state value; the exposure protocol and testing state must therefore accompany any reported strength [94]. For particleboard and fiberboard, thickness swelling and internal bond strength provide additional information on panel performance [95,96]. Reduced water absorption and swelling in lignin–oxidized starch particleboards demonstrate improved panel-level moisture response, but do not isolate the water resistance of the adhesive bondline [97].

For bonded wood structures exposed to prolonged moisture or outdoor conditions, strength measured after a single water-immersion treatment is insufficient to comprehensively assess their reliability in service. Load-bearing capacity, delamination, and cumulative damage following repeated wetting–drying cycles must also be considered. The evaluation of structural wood adhesives must also account for requirements related to service environments and bondline durability. For example, EN 301 specifies classification and performance requirements for phenolic and aminoplastic adhesives used in load-bearing timber structures [98], whereas EN 15425 establishes corresponding requirements for one-component polyurethane adhesives used in such structures [99]. Zhang et al. [100] developed poly(vinyl alcohol)–lignin adhesive films for plywood bonding. The resulting plywood joints exhibited a bond strength of 2.13 MPa after 3 h of hot-water treatment at 63 ± 3 °C and retained a strength of 1.07 MPa after 10 wetting–drying cycles.

### 4.2. Moisture Response and Structural Stability of Cured Lignin-Based Adhesive Networks

The water resistance of cured lignin-based adhesive networks depends on the distribution of hydrophilic functional groups, the nature of intermolecular linkages, and the spatial continuity of the network. Hydroxyl, amino, and ionic groups in lignin and its blended components can interact with water molecules through hydrogen bonding or hydration, whereas covalent linkages within the network can restrict chain segment expansion and network swelling upon water uptake [101,102]. Yu et al. [102] subjected lignosulfonate to phenolation and subsequent epoxidation to prepare phenolated lignin epoxy resin (PLEP), which was then combined with soy protein to formulate a composite adhesive. Based on chemical structural characterization and molecular dynamics simulations, the authors associated enhanced intermolecular bonding with covalent linkages, hydrogen bonding, π–π interactions, and ionic coordination within the system. The optimized formulation exhibited a residual fraction of 81.22% after water immersion and a wet shear strength of 2.07 MPa. These findings suggest that lignin-based systems retaining polar functional groups can nevertheless achieve relatively high structural stability upon water immersion through covalent network formation and multiple intermolecular interactions.

**Figure 6:**
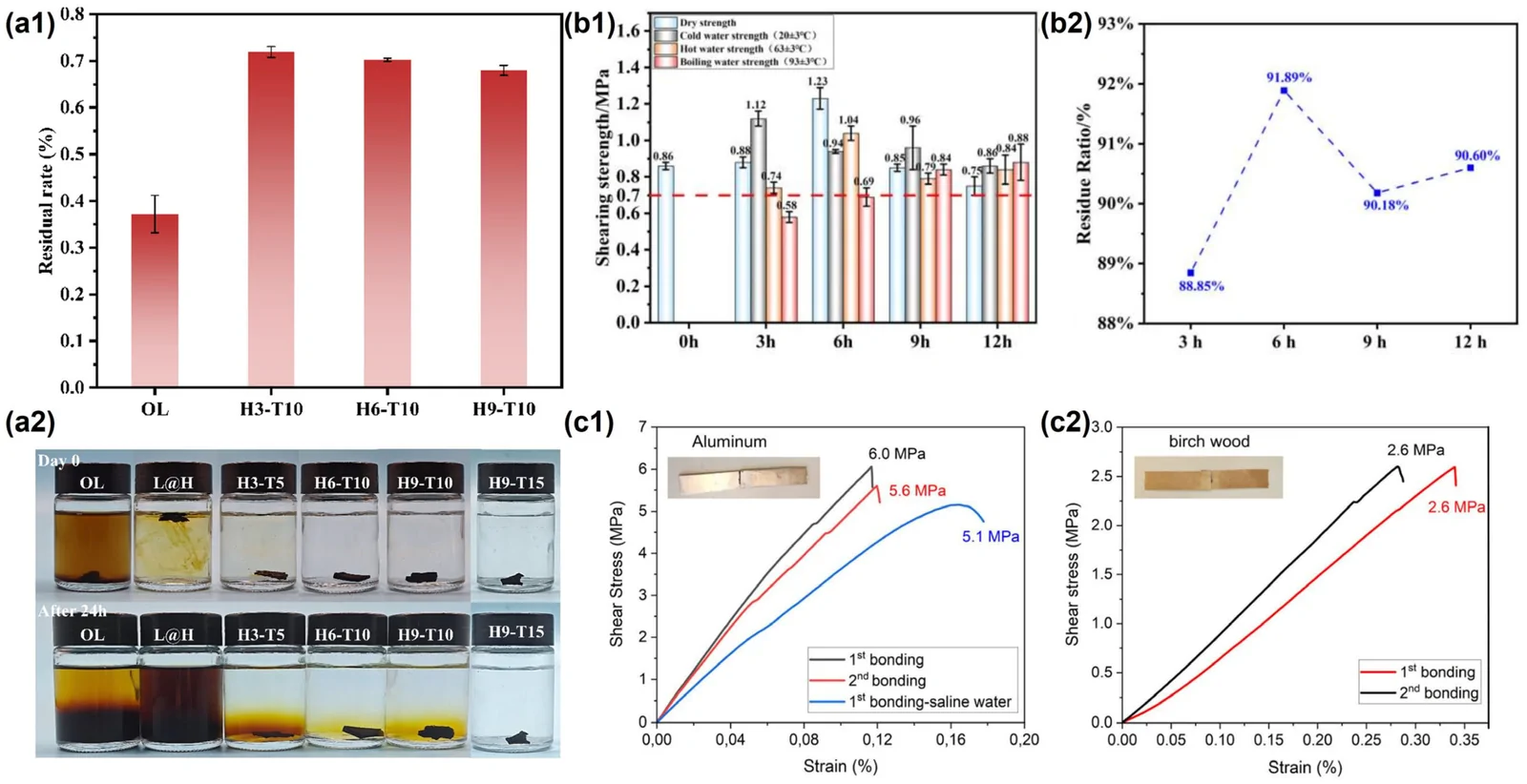
Aqueous stability and bonding performance of lignin-based cured networks. (**a1**,**a2**) Effect of crosslinking formulation on the aqueous stability of cured lignin-based materials. Reprinted from Ref. [101]: (**a1**) residual mass fractions of oxidized lignin and different crosslinked formulations after hot-water treatment at 63 °C for 6 h; (**a2**) appearance of different formulations before and after 24 h of water immersion. (**b1**,**b2**) Effect of starch oxidation time on the water resistance of oxidized starch–lignin adhesives. Reprinted from Ref. [29]: (**b1**) shear strengths under dry conditions and after cold-water, hot-water, and boiling-water treatments; (**b2**) residual mass fractions of the corresponding cured adhesives after water treatment (The red dashed line in (**b1**) indicates the 0.7 MPa reference threshold used in the original study. The blue dashed line in (**b2**) connects the measured values as a guide to the eye.). (**c1**,**c2**) Water-resistant bonding and rebonding performance of lignin-based dyna mic acetal networks. Reprinted from Ref. [103]: (**c1**) shear stress–strain curves for initial bonding, rebonding, and initial bonding followed by saline-water immersion on aluminum substrates; (**c2**) shear stress–strain curves for initial bonding and rebonding on birch substrates. Rebonding was performed by hot pressing at 150 °C for 2 h. Saline-water treatment consisted of immersion in a 32 wt% saline solution for 100 h and applied only to the aluminum-bonded specimens.

The ability of effective network linkages to restrict swelling can be examined by varying the proportions of crosslinking components. In the HBPA-functionalized lignin/epoxy system, crosslinking increased mass retention after hot-water exposure, whereas a formulation containing excess amino groups exhibited greater swelling [101] (Figure 6(a1,a2); Table 3). At a near-constant HBPA-to-epoxy mass ratio, increasing the reactive-component loading reduced swelling and increased the apparent crosslinking density estimated from the swelling model. These results emphasize the balance between network-forming reactions and residual hydrophilic groups: adding reactive functionality is beneficial only when its effective incorporation into the network is considered.

The degree of activation of reactive components can also influence the ability of cured materials to maintain their structural integrity in water. Liu et al. [29] oxidized starch using ammonium persulfate and subsequently blended it with lignin to prepare oxidized starch–lignin adhesives. They compared the bonding strength and residual fraction of the cured materials at different starch oxidation times (Figure 6(b1,b2)). As the starch oxidation time increased from 3 to 6 h, the residual fraction after water treatment increased from 88.85% to 91.89%, accompanied by an increase in bond strength after hot-water treatment. Further prolonging the oxidation time resulted in lower residual fractions and post-treatment bond strengths than those obtained with the 6 h formulation. The authors associated these changes with the formation and subsequent transformation of reactive functional groups during oxidation, suggesting that the degree of starch activation influences the water stability of the cured network by modifying its reactivity toward lignin.

Incorporating flexible chain segments to tailor network structure can also alter deformation constraints and mechanical responses following water uptake. Polyacrylamide-grafted lignin combined with an epoxy crosslinker provides an example in which flexible segments and covalent connectivity were introduced together [104]. The accompanying improvements in moisture uptake, mass retention, and wet strength are summarized in Table 3. Compared with the formulation-balance approach discussed above [101], this strategy also changes the connecting segments within the network. Together, these studies suggest that the moisture stability of lignin-based bondlines depends not only on the number of network linkages but also on the chemical nature of the connecting segments and the constraints they impose on molecular mobility.

Oxidative activation of lignin aromatic structures provides an alternative reaction pathway for constructing water-resistant networks. Liu et al. [106] employed demethoxylation to obtain catechol-rich lignin and subsequently incorporated Cu^2+^ and soy protein isolate (SPI) to construct a network involving covalent linkages, hydrogen bonding, and metal coordination. The resulting adhesive achieved a wet shear strength of 1.29 MPa, representing a 92.5% increase compared with the control formulation. In such systems, reactions between quinone groups and nucleophilic functional groups in proteins can establish relatively stable linkages, whereas hydrogen bonding and coordination interactions influence local molecular associations and network organization. These findings suggest that an increase in phenolic functional groups is not the sole factor contributing to improved water resistance; their oxidation state, reactivity toward proteins, and the types of linkages formed also influence wet-state performance.

Physical associations within multicomponent networks are also influenced by component proportions and compatibility. In soy protein adhesives containing lignin and PAE, low modifier loadings improved wet bonding, whereas further additions reduced spreadability without providing additional strength gains [105] (Table 3). This non-monotonic response shows that formulation changes must preserve adhesive application and contact as well as promote network interactions.

Spatial heterogeneity within the network can further influence the pathways through which moisture penetrates cured materials. For lignin-based epoxy polymers, Kafashan et al. [107] prepared epoxy coatings using kraft lignin fractions of different molecular weights and investigated water uptake and diffusion behavior through gravimetric mass-gain measurements and electrochemical impedance spectroscopy (EIS). The results showed that the low-molecular-weight lignin fraction obtained by ethyl acetate fractionation exhibited better dispersion and compatibility within the epoxy matrix, resulting in lower equilibrium water uptake and a lower diffusion coefficient. In contrast, aggregation and poorer compatibility of higher-molecular-weight lignin were associated with increased water uptake. Although this study focused on lignin-based epoxy coatings rather than wood-bonded joints, its findings on moisture transport in polymeric materials provide insight into how component aggregation and compatibility may influence moisture migration pathways. Considered alongside studies of cured morphology in lignin–protein adhesives, these findings suggest that network continuity, local phase distribution, and intermolecular linkages collectively influence moisture ingress and transport within cured materials.

Network stability under moisture exposure also involves reversible intermolecular interactions and dynamic covalent linkages. Liu et al. [108] employed ultrasonic treatment to introduce catechol structures into lignin and subsequently combined the modified lignin with soy protein to construct a composite network involving both dynamic covalent bonds and hydrogen bonding. The authors proposed that imine linkages formed between quinone-derived structures and amino groups in the protein contributed to improved wet bonding performance, while the hydrogen-bonding network facilitated energy dissipation. The modified adhesive achieved a wet shear strength of 1.37 MPa and could be reused three times through network reorganization. Moreno et al. [103] constructed a dynamic acetal network from softwood kraft lignin and poly(ethylene glycol) divinyl ether and characterized its thermally triggered network rearrangement through stress relaxation and reprocessing tests. The material exhibited an initial lap-shear strength of 6.0 MPa on aluminum substrates. Following failure, the specimens were rebonded by hot pressing at 150 °C for 2 h, achieving a lap-shear strength of 5.6 MPa. A separate set of specimens retained a bond strength of 5.1 MPa after immersion in a 32 wt% saline solution for 100 h (Figure 6(c1)). On birch wood substrates, both the initial and rebonded joints exhibited bond strengths of approximately 2.6 MPa (Figure 6(c2)). The saline-immersion and rebonding results demonstrate, respectively, the ability of this network to retain bonding performance under the investigated aqueous conditions and its capacity for repeated bonding enabled by thermally triggered network rearrangement.

For adhesives required to maintain their mechanical performance under elevated temperature and humidity, restrictions on polymer chain segmental mobility are as important as network connectivity. Li et al. [109] incorporated hydroxymethylated lignin into poly(vinyl acetate) (PVAc) systems and employed diphenylmethane diisocyanate (MDI) to construct interpenetrating and semi-interpenetrating polymer networks. The resulting modified adhesive exhibited a bond strength of 1.8 MPa after hot-water treatment. Unlike lignin–protein systems that rely on dynamic imine linkages and hydrogen-bond reorganization, this system employs a crosslinked network to restrict the segmental mobility of PVAc chains, thereby improving bonding performance after hot-water treatment. Together, these studies indicate that the moisture stability of cured adhesive networks depends not only on the formation of continuous structures through crosslinking reactions but also on their ability to maintain constraints on segmental mobility and load-bearing capacity under moisture uptake and temperature variations.

Overall, existing studies have established relationships between the structure and moisture response of lignin-based adhesives at multiple levels. Reaction-derived linkages can increase the insoluble fraction and restrict swelling in certain systems, while component dispersion and compatibility influence moisture transport pathways. Flexible chain segments and dynamic intermolecular interactions can further modify the mechanical response of the network following water uptake. Studies combining measurements of residual material after water immersion, swelling tests, and analyses of apparent crosslinking density provide more direct evidence of network structural stability. In contrast, studies relying solely on wet bond strength measurements and dry-state structural characterization primarily reveal associations between formulation structure and bonding performance. Accordingly, the water resistance of cured lignin-based adhesive networks can be understood in terms of the combined effects of water affinity, effective network connectivity, structural homogeneity, and segmental mobility. The specific contributions of these factors must be evaluated in relation to the chemical composition and moisture-response data of each adhesive system.

### 4.3. Wet-State Mechanical Response and Failure Mechanisms at the Wood–Adhesive Interface

**Figure 7:**
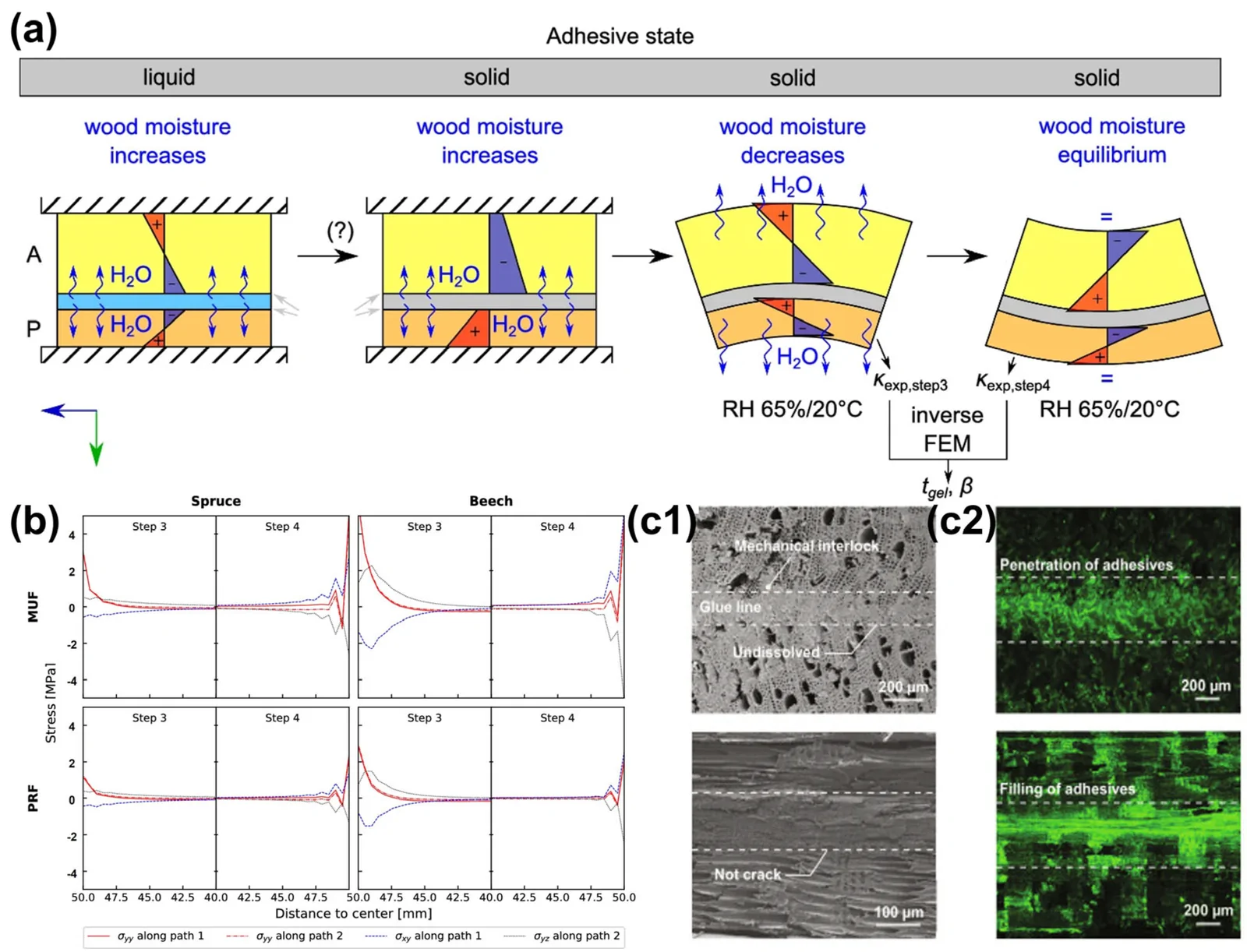
Residual stresses, interfacial morphology, and microstructural defects in wood–adhesive bondlines. (**a**) Schematic illustration of residual deformation induced by moisture migration, adhesive curing, and constrained deformation of wood layers during bonding. Reprinted from Ref. [111]. The yellow and orange regions represent the active (A) and passive (P) wood layers, respectively. Blue arrows labeled H_2_O indicate moisture transport, and black arrows connecting the stages indicate process progression. Red “+” and blue “−” symbols indicate tensile and compressive stresses, respectively. (**b**) simulated stress distributions in the adhesive bondline for different wood–adhesive combinations during unloading and moisture re-equilibration. Reprinted from Ref. [111]; (**c1**,**c2**) interfacial morphology and spatial distribution of lignosulfonic acid–citric acid adhesive in bonded wood. Reprinted from Ref. [112]: (**c1**) scanning electron microscopy (SEM) images of the bondline region after boiling-water treatment and (**c2**) confocal laser scanning microscopy (CLSM) images, in which green fluorescence shows the adhesive distribution within the bondline and adjacent wood tissues.

Anisotropic moisture-induced swelling of wood, together with the constraints imposed by adhesive bonding, is an important source of local stresses during changes in moisture conditions. Residual stresses generated during joint fabrication also establish the initial stress state for subsequent loading. Digital image correlation measurements showed that humidity-dependent differences between earlywood and latewood altered strain distribution and failure location in bonded joints [110]. Complementary bending experiments and finite element analysis linked residual deformation to moisture transfer during curing and subsequent moisture re-equilibration [111] (Figure 7a,b). Together, these findings distinguish moisture-induced stresses developed during joint fabrication from those generated by later humidity changes. Wet-state failure should therefore be interpreted in relation to both the initial stress state established during curing and the subsequent moisture response of the bonded wood.

The integrity of near-surface wood tissues and the effective contact established by the adhesive jointly influence bonding performance after moisture exposure. Knorz et al. [113] compared joints prepared from ash wood surfaces subjected to planing, sanding, or end milling and bonded with phenol–resorcinol–formaldehyde (PRF), melamine–urea–formaldehyde (MUF), polyurethane (PUR), and emulsion polymer isocyanate (EPI) adhesives. They found that shear and delamination test results varied with both surface preparation method and adhesive type. Microscopic observations revealed differences in near-surface cell damage, the degree of surface fibrillation, and surface morphology resulting from different machining methods, with their effects on moisture resistance depending on the adhesive system. Masoumi et al. [114] further observed that wood anatomical structure influenced local adhesive distribution in different cross-laminated timber (CLT) configurations, with different penetration characteristics occurring on opposite sides of bondlines joining different wood species. Together, these studies link machining-induced surface conditions with the intrinsic porous structure of wood, indicating that wet bonding performance depends on the interfacial bonding characteristics established between a given wood surface and adhesive. The effects of surface preparation should therefore be evaluated in relation to the specific adhesive system.

Introducing reactive sites onto wood surfaces can enhance chemical bonding between lignin-based adhesives and the substrate, thereby improving interfacial bonding under wet conditions. Yuan et al. [115] formulated a composite adhesive using quinone-functionalized lignin, aminated cellulose, and epoxidized lignin. A multiply crosslinked network was constructed through reactions between quinone moieties and amino groups, together with epoxy–amine ring-opening addition reactions. The wood surface was further functionalized with amino groups using 3-aminopropyltriethoxysilane (APTES). With the same adhesive formulation, joints prepared from activated wood exhibited a dry shear strength of 3.20 MPa and a wet shear strength of 1.73 MPa after immersion in water at 63 °C for 3 h, whereas untreated wood joints exhibited no measurable wet bond strength under the same conditions. The authors attributed this improvement to enhanced covalent bonding between the adhesive and the activated wood surface. Liu et al. [112] converted sodium lignosulfonate into lignosulfonic acid through ion exchange and utilized its acid-catalytic activity to promote esterification reactions involving citric acid, thereby preparing a lignin-based adhesive. The authors employed scanning electron microscopy (SEM) and confocal laser scanning microscopy (CLSM) to characterize the morphology of the wood bonding region and the spatial distribution of the adhesive. SEM images of transverse and longitudinal sections of the plywood after boiling-water treatment showed that the adhesive filled some wood pores and formed a continuous bonding region, with no obvious interfacial separation observed in the examined areas (Figure 7(c1)). CLSM images further revealed the spatial distribution of the adhesive within the bondline and adjacent wood tissues (Figure 7(c2)). Whereas Yuan et al. enhanced reactive-site availability through amination of the wood surface, Liu et al. promoted network formation and interfacial bonding by modifying the reaction environment during curing. These two strategies illustrate the respective contributions of substrate surface activation and curing reaction control to wet bonding performance.

The balance between effective adhesive contact within wood pores and adhesive retention within the bondline further influences the continuity of the cured bondline and the distribution of defects. Zhang et al. [116] used lignin colloids to modify a water-in-oil isocyanate emulsion. The colloids increased the apparent viscosity of the continuous isocyanate phase, thereby suppressing excessive adhesive penetration. They also formed a colloidal film at the isocyanate–water interface, limiting direct contact between the two phases and delaying premature reactions. Following modification, the adhesive penetration depth into wood decreased from approximately 200 to 25 μm, and the cured bondline exhibited improved continuity. Meanwhile, the dry shear strength increased from 1.02 to 1.74 MPa, and strength retention after boiling-water treatment reached 93%. Masoumi et al. [114] used optical microscopy to examine bondlines in different cross-laminated timber (CLT) specimens and observed microscopic defects, including voids and gaps, within the adhesive layer and near the wood–adhesive interface. They further employed image analysis to compare defect distributions and area fractions among the specimens. Their results showed that greater effective adhesive penetration, thinner bondlines, and fewer defects were generally associated with higher shear strength and reduced delamination. Together, these studies indicate that adhesive penetration must balance effective contact within wood pores with sufficient adhesive retention in the bondline. Bondline thickness, defect distribution, and structural continuity after curing collectively influence the load-bearing performance of bonded wood structures.

Post-peak deformation and energy dissipation during crack propagation provide mechanical information beyond shear strength for evaluating the load-bearing behavior of bonded joints after moisture exposure. van Blokland and Serrano [117] characterized the traction–separation relationships of birch wood joints bonded with bio-based adhesives using dry-state Mode I fracture tests. The lignin-based adhesive joints exhibited a peak traction of approximately 3 MPa and a specific fracture energy of approximately 110 J·m^−2^, compared with approximately 4 MPa and 370 J·m^−2^, respectively, for the fish-glue-bonded joints. The steeper post-peak softening response and lower fracture energy of the lignin-based joints highlight the distinction between peak load-bearing capacity and energy dissipation during crack propagation. Raftery et al. [118] employed double cantilever beam (DCB) tests to compare the Mode I fracture performance of bonded engineered-timber joints after accelerated aging. They found that the investigated swelling–shrinkage cycles did not cause a statistically significant reduction in the Mode I fracture performance of joints bonded with one-component polyurethane (1C-PUR) or resorcinol–formaldehyde (RF) adhesives. In contrast, prolonged aging under elevated temperature and humidity significantly reduced the performance of certain joints made from preservative-impregnated wood, which the authors considered more likely to result from weakening of the wood substrate. Feng et al. [119] further coupled moisture transport and delamination models for wood structures with externally bonded fiber-reinforced polymer (FRP) reinforcement. They investigated the combined effects of local moisture-induced swelling stresses and interfacial fracture parameters on damage evolution as the adhesive layer altered the moisture distribution. Collectively, these studies demonstrate, from the perspectives of joint traction–separation behavior, aging-induced changes in the substrate, and coupled moisture–mechanical responses, that fracture performance should be interpreted in relation to moisture exposure history, the region of crack propagation, and structural constraints.

Delamination behavior following repeated moisture exposure reflects the ability of bonded structures, formed through the combined effects of adhesive formulation and wood surface conditions, to maintain their integrity. Ayanleye et al. [120] incorporated fractionated lignin into polyurethane (PUR) adhesives at addition levels of 1%, 2%, and 3% for the fabrication of engineered timber products. They evaluated block shear strength, wood failure percentage, and delamination performance. All lignin-modified formulations met the ASTM D2559 delamination requirements applied in the study. The formulation containing 1% lignin exhibited the lowest delamination percentage of 0.07% and showed better overall bonding performance than the formulations containing 2% and 3% lignin. These studies identify lignin addition level and the combination of surface preparation method and adhesive type as formulation-related and substrate-related factors, respectively, affecting bonding performance after moisture exposure.

## 5. Multiscale Structure–Property Relationships Between Cured Network Structures and Water Resistance in Lignin-Based Wood Adhesives

### 5.1. Effects of Reactive-Site Utilization and Curing Progression on Wet Bonding Performance

**Figure 8:**
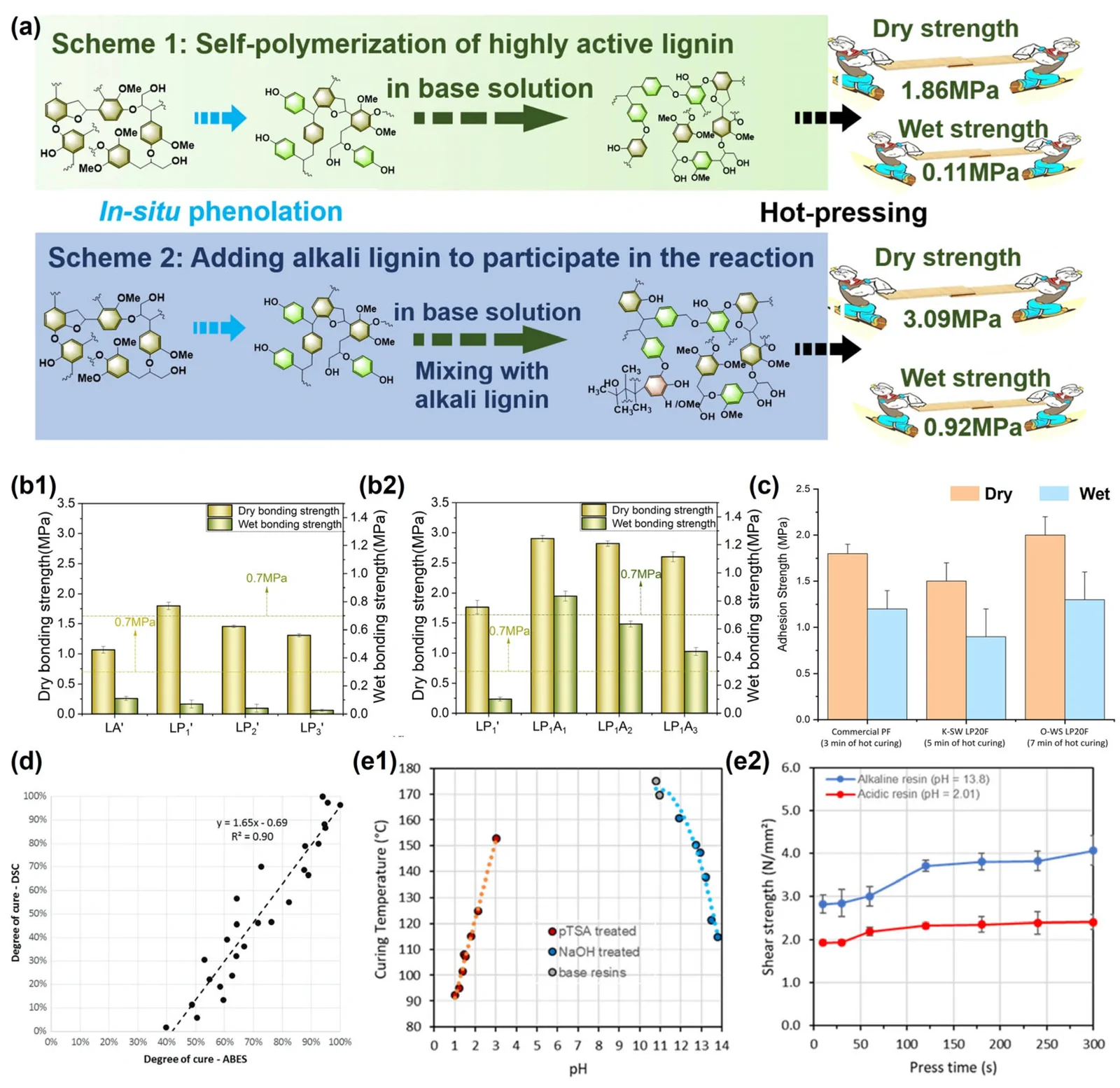
Relationships among precursor composition, curing conditions, and bonding performance of lignin-based adhesives. (**a**) Schematic illustration of the reaction pathways and representative dry and wet bonding strengths of in situ phenolated lignin used alone or blended with alkali lignin. Reprinted from Ref. [121]. (**b1**,**b2**) Effect of lignin composition on bonding performance. Reprinted from Ref. [121]: (**b1**) dry and wet bonding strengths of alkali lignin and different in situ phenolated lignins used individually; (**b2**) dry and wet bonding strengths of in situ phenolated lignin blended with different amounts of alkali lignin. (**c**) Dry and wet bonding strengths of commercial PF, kraft lignin-based phenolic resin (K-SW LP20F), and organosolv lignin-based phenolic resin (O-WS LP20F) after hot pressing for 3, 5, and 7 min, respectively. Reprinted from Ref. [123]. (**d**) Correlation between the degrees of cure determined by DSC and ABES for a lignin-based NIPU adhesive. Reprinted from Ref. [87]. (**e1**,**e2**) Effect of reaction-medium pH on the curing behavior and strength development of kraft lignin–5-hydroxymethylfurfural adhesives. Reprinted from Ref. [124]: (**e1**) relationship between pH and curing temperature determined by DSC; (**e2**) shear strength development with pressing time at 130 °C for formulations at pH 13.8 and 2.01.

The contribution of lignin functional-group activation to wet bonding performance depends on the extent to which the activated structures form linkages during curing and how the resulting network responds to moisture. Hu et al. [121] increased the phenolic hydroxyl content of lignin through in situ phenolation and compared its bonding performance when used alone or blended with alkali lignin (Figure 8a,(b1,b2)). When used alone, the in situ phenolated lignin adhesive achieved a maximum dry bond strength of 1.86 MPa, but none of the formulations passed the wet bonding test. The authors attributed this discrepancy to the increased affinity for water associated with the abundant hydroxyl groups introduced by phenolation (Figure 8(b1)). After blending with alkali lignin, the optimized formulation achieved dry and wet bond strengths of 2.95 and 0.86 MPa, respectively (Figure 8(b2)). Further optimization of the hot-pressing conditions increased these values to 3.09 and 0.92 MPa, respectively (Figure 8a). Chemical characterization before and after curing provided evidence of increased intermolecular linkages between lignin units and a greater abundance of ether-linkage-related structures. These findings suggest that the reactivity introduced by precursor activation must be complemented by appropriate blend composition and hot-pressing conditions to translate into load-bearing capacity after water exposure. Sepahvand et al. [122] selectively demethylated technical lignin using iodocyclohexane, reducing its methoxy group content, increasing its phenolic hydroxyl content, and narrowing its molecular weight distribution. At a phenol substitution level of 50%, panels bonded with the modified lignin–phenol–formaldehyde resin exhibited an internal bond strength of 1.03 MPa after boiling-water treatment, compared with 0.45 MPa for the unmodified LPF50 system. Together, these studies demonstrate that an increase in phenolic reactive sites does not necessarily translate directly into improved wet bonding performance. Its effects are also governed by lignin structural distribution, blend composition, and the conditions under which network linkages form during curing.

The identity and proportions of co-reactive components further influence the pathways through which lignin reactive sites participate in network formation, thereby affecting how precursor reactivity translates into wet bonding performance. Tao et al. [125] partially replaced phenol and formaldehyde with acetonitrile-extracted lignin and furfural, respectively, and prepared the adhesive through staged co-condensation. At phenol and formaldehyde substitution levels of 40% and 15%, respectively, the formulation achieved a wet tensile bond strength of 1.68 MPa, exceeding the 0.66 MPa obtained with the control resin prepared from technical lignin. These results link differences in bonding performance to lignin feedstock selection and co-condensation formulation, highlighting the combined roles of precursor structure and co-reactant design. Nafisah et al. [126] partially replaced phenol with 10–30% lignin in a room-temperature-curing phenol–resorcinol–formaldehyde (PRF) resin system and reported improvements in resin reactivity, the development of methylene-bridge-related structures, and the dry and wet bonding performance of laminated veneer lumber (LVL). In contrast to the heat-cured system incorporating furfural, the PRF system forms network linkages under different phenolic-component compositions and curing conditions. This comparison indicates that the utilization of lignin reactive sites is jointly governed by feedstock functional-group composition, co-reactive components, and condensation conditions.

The relationship between chemical reaction progress and the development of mechanical properties in bonded joints is system-dependent. Accordingly, establishing a correspondence between the degree of cure determined by thermal analysis and actual bond strength development is essential for linking curing behavior to wet bonding performance. Siahkamari et al. [123] prepared phenol–formaldehyde adhesives incorporating kraft lignin or organosolv lignin at a phenol substitution level of 80%. They found that sodium carbonate, used as a cocatalyst, reduced the characteristic curing temperatures and apparent activation energy. Nevertheless, sufficient hot-pressing time was still required to meet the specified wet wood failure percentage criterion. After hot pressing for 3, 5, and 7 min, respectively, the commercial PF, kraft lignin-based, and organosolv lignin-based systems exhibited dry bond strengths of approximately 1.8, 1.5, and 2.0 MPa and wet bond strengths of approximately 1.2, 0.9, and 1.3 MPa (Figure 8c). In the same study, extending the hot-pressing time from 3 to 7 min increased the wet wood failure percentage from 80% to 100% for the kraft lignin-based system and from 60% to 90% for the organosolv lignin-based system. These results indicate that improvements in curing characteristics identified by thermal analysis must be accompanied by an appropriate hot-pressing schedule to achieve the desired wet bonding performance. Saražin et al. [87] further characterized the chemical degree of cure using normalized reaction enthalpy and the mechanical degree of cure using normalized bond strength measured by the Automated Bonding Evaluation System (ABES). For the lignin-based non-isocyanate polyurethane (NIPU) adhesive, linear regression between these two measures yielded an R^2^ value of approximately 0.90 (Figure 8d). Despite this strong correlation, the DSC-derived chemical degree of cure was generally lower than the ABES-derived mechanical degree of cure over the investigated curing intervals. This finding suggests that chemical conversion is closely related to bond strength development, although the quantitative correspondence between the two remains dependent on the characterization method and bonding conditions.

The reaction medium can also simultaneously alter the thermal curing response and the dispersion state of lignin components, leading to different trends in curing temperature and bond strength development. Dorn et al. [124] investigated kraft lignin–5-hydroxymethylfurfural (HMF) adhesives and found that the curing temperature determined by differential scanning calorimetry (DSC) was below 100 °C under strongly acidic conditions, compared with approximately 115 °C at a pH of around 13.8 (Figure 8(e1)). At the same solids content and a pressing temperature of 130 °C, the alkaline formulation exhibited higher shear strength measured by the Automated Bonding Evaluation System (ABES) than the acidic formulation at pH 2.01 over the corresponding pressing times (Figure 8(e2)). The authors also observed that the resin tended to aggregate under acidic conditions, producing a heterogeneous mixture, which they attributed to reduced lignin solubility. By linking the reaction medium, component dispersion, and bond strength development, this study demonstrates that a lower curing temperature determined by thermal analysis should be evaluated alongside adhesive formulation stability and load-bearing performance under the intended processing conditions.

The performance of low-temperature-curing formulations depends on the extent to which curing reactions proceed and effective networks form under the intended hot-pressing conditions, while the curing onset temperature and apparent activation energy characterize different aspects of this process. The aminated-lignin/tannin NIPU formulation reinforced with MCC provides an example of maintaining wet bonding performance at a reduced curing temperature [55]. Du et al. [127], by contrast, observed a decrease in curing onset temperature accompanied by an increase in the apparent activation energy of thermal curing in sodium lignosulfonate-modified urea–formaldehyde (UF) resins. At an addition level of 1%, the modified formulation achieved a wet bond strength of 1.28 MPa, representing a 23% increase over the unmodified system. These findings indicate that a reduction in curing onset temperature does not necessarily coincide with a decrease in apparent activation energy, and that wet bonding performance under the intended processing conditions must also be evaluated based on the actual load-bearing performance of the cured joints.

The contact between reactive components links particle-scale processing to molecular-scale crosslinking reactions, thereby influencing the cured network structure and bonding performance. Hafez et al. [128] developed heat-cured adhesives from lignin-rich saccharification residues and found that wet milling reduced particle size and improved bond strength. When the residue was used as the sole adhesive component, the dry single-lap shear strength reached 6.25 MPa, approximately 80% of that of the commercial phenol–formaldehyde (PF) control examined in the study. When the formulation contained 66.7% saccharification residue and 33.3% citric acid, its dry and wet bonding performance was comparable to that of the PF control. Chemical characterization provided evidence supporting the formation of ester linkages involving components of the saccharification residue. Unlike the approach adopted by Hu et al. [121], who modified bonding performance through lignin activation and blending, this study linked feedstock processing to subsequent crosslinking by improving contact between reactive components through particle size reduction and reinforcing the cured structure through linkages involving a polycarboxylic acid. Considered alongside studies of hot-pressing time and comparisons between differential scanning calorimetry (DSC) and the Automated Bonding Evaluation System (ABES), these findings provide a clearer understanding of curing–performance relationships. Particle-level contact and co-reactive components influence linkage formation, chemical curing and bond strength development do not necessarily proceed in parallel, and extended hot-pressing times can further alter the load-bearing capacity and failure characteristics of bonded joints after water exposure.

### 5.2. Effects of Crosslinked Networks and Wood–Adhesive Interfaces on Water Resistance

The load-bearing capacity of lignin-based adhesives under wet conditions is jointly influenced by the crosslinked structure of the adhesive layer, adhesive distribution within wood pores, and the nature of interfacial bonding. These effects can be analyzed by correlating chemical structural characterization with wet bond strength and failure morphology [56,129,130].

Lignin-derived epoxy components can form adhesive networks capable of sustaining loads under wet conditions through reactions with different co-reactive components. Zhao et al. [131] subjected alkali lignin to demethylation and nanosizing, followed by epoxidation, and subsequently cured the resulting epoxy components through epoxy–amine reactions. The bonded wood joints exhibited a dry shear strength of 2.45 ± 0.12 MPa and retained a shear strength of 2.24 ± 0.15 MPa after 72 h of cold-water immersion, corresponding to a strength retention of 91.3%. Changes in Fourier-transform infrared (FTIR) spectra supported the participation of reactive functional groups in network formation. Xiong et al. [132] prepared lignin-based epoxy resins through sequential oxidation and epoxidation and blended them with starch. The resulting plywood exhibited dry and wet shear strengths of 1.66 ± 0.25 and 0.99 ± 0.18 MPa, respectively. The consumption of epoxy groups and changes in absorption bands associated with oxygen-containing linkages after curing were used by the authors to support a proposed mechanism involving covalent bond formation between the lignin-derived epoxy components and starch. These two studies illustrate distinct pathways for connecting lignin-derived epoxy structures through amine curing agents and polyhydroxyl components.

**Figure 9:**
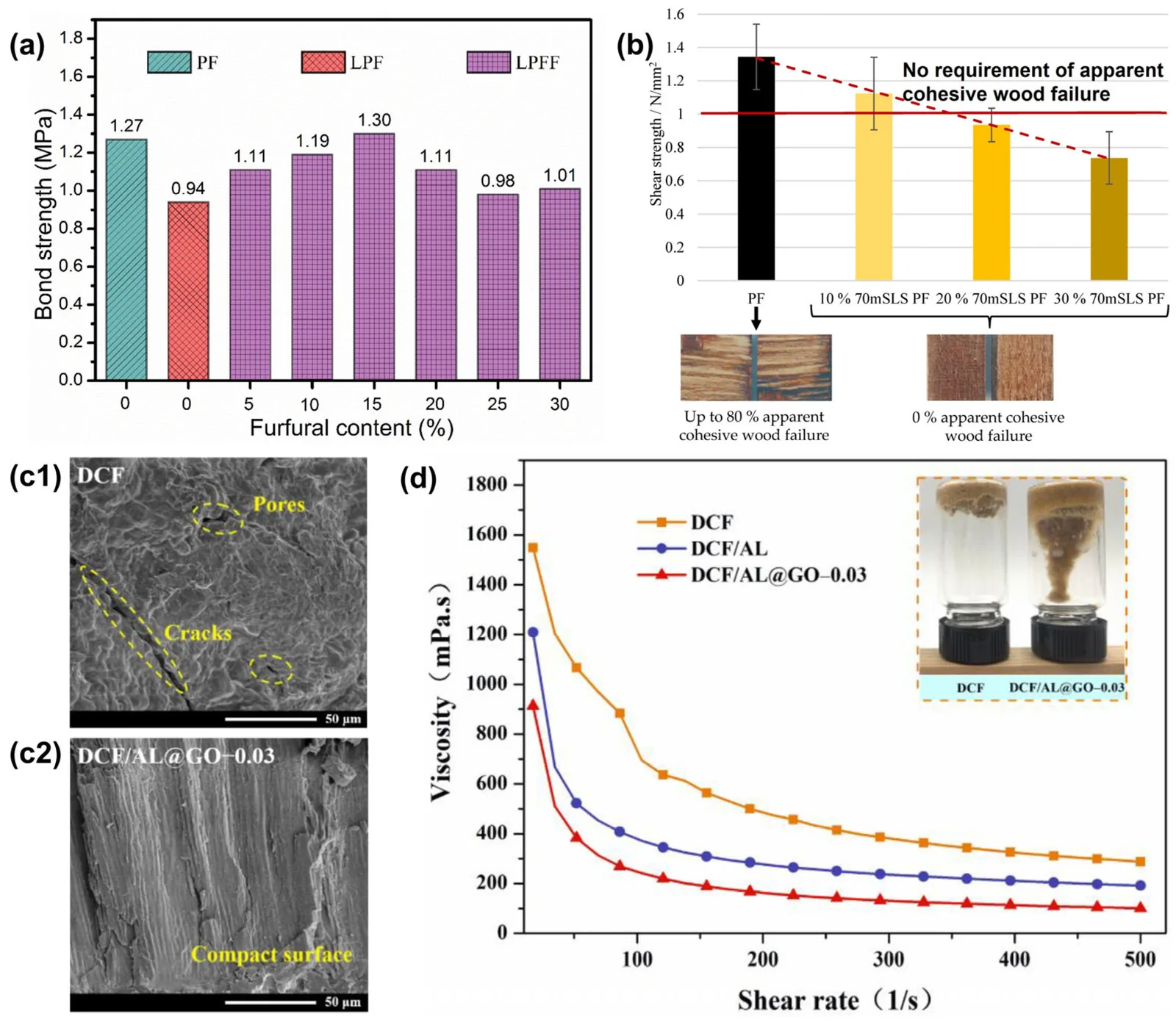
Formulation-dependent bonding performance, cured adhesive morphology, and rheological behavior of lignin-based wood adhesives. (**a**) Shear strengths of lignin–phenol–formaldehyde adhesives containing different amounts of furfural after two boiling-water treatments with intermediate drying; PF and LPF denote the phenol–formaldehyde and furfural-free lignin–phenol–formaldehyde controls, respectively. Reprinted from Ref. [133]. (**b**) Shear strengths and representative wood-failure appearances of plywood bonded with resins containing different phenol replacement levels of methylolated softwood lignosulphonate after EN 314 bonding class 3 pretreatment; the red horizontal line marks the 1 N·mm^−2^ threshold above which no apparent cohesive wood-failure requirement applies. Reprinted from Ref. [134]. (**c1**,**c2**) SEM images of cured cottonseed-based adhesive fracture surfaces. Reprinted from Ref. [135]: (**c1**) defatted cottonseed flour (DCF) control and (**c2**) alkali lignin/graphene-oxide-modified formulation DCF/AL@GO-0.03. (**d**) Viscosity–shear rate curves of DCF, DCF/AL, and DCF/AL@GO-0.03 adhesives; the inset shows inverted-vial photographs of DCF and DCF/AL@GO-0.03. Reprinted from Ref. [135].

The effects of co-reactive component content and lignin substitution level on water resistance can be evaluated through formulation comparisons within individual adhesive systems. Zhang et al. [133] investigated furfural-modified lignin–phenol–formaldehyde resins and found that, at a furfural addition level of 15%, the plywood shear strength after two boiling-water treatments with intermediate drying increased from 0.94 MPa for the LPF control to 1.30 MPa. Further increases in furfural content resulted in decreased shear strength (Figure 9a). Gonçalves et al. [134] found that increasing the phenol substitution level of hydroxymethylated softwood lignosulfonate from 10% to 30% progressively reduced the plywood shear strength after pretreatment for exterior bonding quality assessment. The corresponding formulations all exhibited apparent cohesive wood failure percentages of 0% (Figure 9b). These two studies reveal the respective effects of co-reactive component addition and lignin substitution level on wet bonding performance, indicating that formulation optimization requires consideration of both network formation and the load-bearing and failure characteristics of bonded joints after water exposure.

The chemical characteristics of co-reactive components further influence how lignin is incorporated into composite networks and the structural roles it performs. Wu et al. [129] modified alkali lignin with multifunctional epoxy compounds to obtain epoxidized lignin (EPL), which was subsequently incorporated into soy protein isolate (SPI) adhesives. The two optimized formulations achieved wet shear strengths of 0.94 and 1.45 MPa, respectively, both substantially exceeding the 0.59 MPa obtained with the unmodified SPI adhesive. The modified systems also exhibited improved hydrophobicity and thermal stability. Zhu et al. [135] incorporated alkali lignin and graphene oxide into defatted cottonseed flour (DCF) adhesives, resulting in a more compact cured fracture surface than that of the DCF control (Figure 9(c1,c2)). The wet shear strength after water immersion at 63 °C for 3 h increased from 0.62 to 1.08 MPa. The combined modification also reduced adhesive viscosity at the same shear rate (Figure 9d), demonstrating its effects on both the structure of the cured adhesive layer and the processing behavior of the adhesive [135].

The balance between rigid and flexible polymeric segments further influences the architecture of covalently linked networks and their wet bonding performance. In the ENR/KH550-functionalized lignin system [56], dry and wet strengths increased and then decreased with increasing modifier content (Table 1). Based on microscopic observations of the failure regions, the authors associated the improved bonding performance with network crosslinking and localized mechanical interlocking between the adhesive and adjacent wood tissues. Shao et al. [130] subjected lignin-containing cellulose nanofibrils (LCNFs) to periodate oxidation to obtain aldehyde-functionalized lignin-containing cellulose nanofibrils (ALCNFs), which were subsequently reacted with aminated poly(vinyl alcohol) (APVA) through Schiff base reactions to construct a rigid–flexible composite network containing dynamic imine linkages. The resulting adhesive achieved a wet shear strength of 1.59 MPa after immersion in water at 63 °C for 3 h. These systems combine flexible polymer segments with lignin-containing structural components through epoxy–amine and imine linkages, respectively.

In lignin-based adhesives, the covalent linkages formed during curing must also be coupled with effective interfacial bonding to contribute to the load-bearing capacity of bonded wood joints. Yang et al. [136] employed a strategy combining hydrodeoxygenation and acid-catalyzed hydroxymethylation to improve the crosslinking potential and flowability during hot pressing of highly condensed technical lignin. Based on structural characterization, the authors proposed that hydroxymethylated lignin formed additional diarylmethane-type covalent linkages during curing, promoting intermolecular bonding within the adhesive layer. Meanwhile, the flowable lignin penetrated wood pores and established mechanical interlocking within the bonding region. Compared with a lignin–phenol–formaldehyde adhesive prepared through conventional alkaline hydroxymethylation, this system exhibited improved bonding performance and water resistance. The authors attributed these improvements to the combined effects of aromatic covalent crosslinking and interfacial mechanical interlocking. Complementary evidence was provided by Yuan et al. [115], who obtained a wet shear strength of 1.73 MPa for amino-functionalized wood surfaces, whereas untreated surfaces exhibited no measurable bond strength with the same adhesive formulation. Together with the mechanical interlocking reported by Yang et al. [136], this comparison links interfacial structure to the wet load-bearing performance of the bonded joint.

### 5.3. Multiscale Structure–Property Relationships Linking Curing, Network Structure, Interfacial Bonding, and Water Resistance

The curing behavior and water resistance of lignin-based wood adhesives involve multiple structural levels, ranging from molecular reactions to load-bearing performance in bonded wood joints. The reactivity of lignin precursors influences the formation of chemical linkages during curing, while the composition and spatial organization of the polymer network further affect bondline cohesion and deformation behavior. Interfacial bonding between the adhesive and wood, in turn, governs the extent to which these structural features contribute to load transfer within the bonded joint [87,97,115]. Existing studies have established structure–property relationships at different levels through functional-group analysis, curing kinetics, network mechanical testing, and evaluations of bonding performance after water exposure. However, these methods probe different material states and structural scales, which are not necessarily directly comparable. Accordingly, the analysis of multiscale structure–property relationships should be grounded in within-system comparisons of structure and performance, with particular attention to distinguishing the respective contributions of molecular structural changes, network formation, and interfacial load transfer to the resulting wet bonding performance.

**Figure 10:**
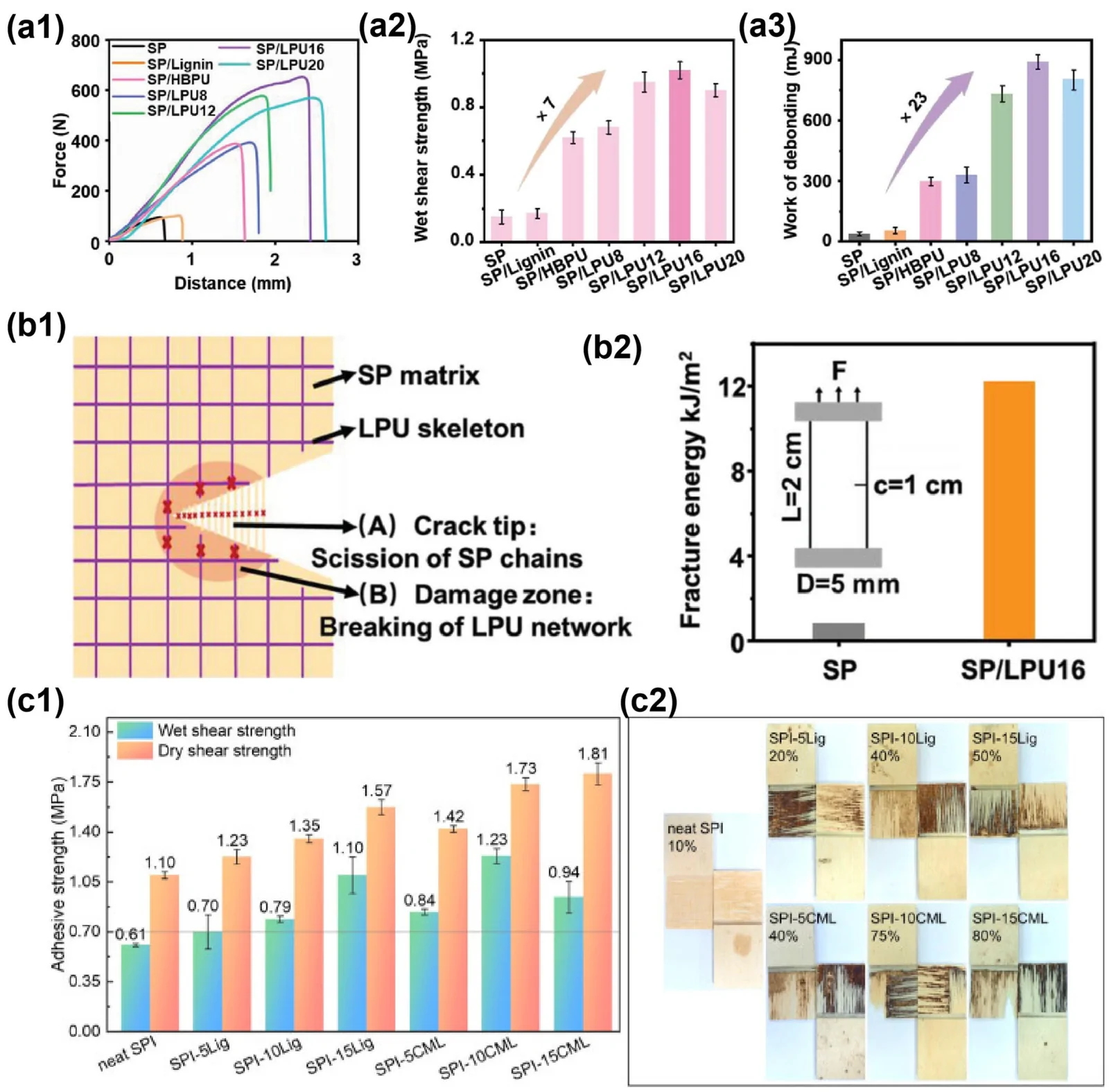
Wet bonding performance, fracture energy dissipation, and failure behavior of lignin-based protein adhesives. (**a1**–**a3**) Force–displacement curves, wet shear strengths, and work of debonding of soybean protein (SP) and lignin-based polyurea/soybean protein (SP/LPU) adhesive joints after treatment in hot water at 63 °C for 3 h. Reprinted from Ref. [138]; (**b1**) proposed mechanism of load transfer and energy dissipation involving the SP matrix and LPU skeleton during crack propagation (The red dotted lines and markers schematically illustrate network rupture near the crack tip and within the surrounding damage zone); (**b2**) fracture energies of cured SP and SP/LPU16 adhesive layers. Reprinted from Ref. [138]; (**c1**,**c2**) dry and wet shear strengths, fracture appearances, and wood failure percentages of plywood bonded with soy protein isolate (SPI) adhesives modified with unmodified lignin (Lig) or carboxymethylated lignin (CML). Reprinted from Ref. [140].

Comparative studies link lignin precursor chemistry and network architecture to differences in curing behavior, wet load-bearing capacity, and fracture response. Yousafzai et al. [137] found that, across different lignin feedstocks, increasing the combined hydroxyl and carboxyl group content from 3.70 to 4.83 mmol·g^−1^ coincided with longer gelation times (360–480 s) and lower shear strengths after hydrothermal treatment (1.92–1.46 MPa). Zhou et al. [138] combined a lignin-based polyurea (LPU) framework with soy protein (SP), increasing wet shear strength from 0.15 to 1.04 MPa and achieving a work of debonding of 902 mJ after hot-water treatment at 63 °C for 3 h (Figure 10(a1–a3)). The authors associated the improved toughness with load transfer through the LPU framework and energy dissipation within the composite network; the fracture energy of the cured adhesive increased from 0.85 to 12.2 kJ·m^−2^ (Figure 10(b1,b2)). Wei and Li [139] likewise reported increased wet strength and reduced moisture uptake after introducing lignin–Fe^3+^ coordination. Sun et al. [140] modified soy protein isolate (SPI) adhesives with carboxymethylated lignin (CML). The SPI-10CML formulation achieved dry and wet shear strengths of 1.73 and 1.23 MPa, respectively, with a wood failure percentage of 75% (Figure 10(c1,c2)). Together, these studies link precursor chemistry and network architecture to complementary measures of wet load bearing, energy dissipation, and failure location.

Differences in the material states and spatial scales probed by various characterization methods represent another important consideration when interpreting these relationships. By comparing differential scanning calorimetry (DSC) and Automated Bonding Evaluation System (ABES) results, Saražin et al. [87] identified a correlation between the chemical degree of cure and bond strength development. However, these processes did not evolve in complete synchrony, and their relationship alone was insufficient to determine joint stability after water exposure. Local nanomechanical investigations of bonded wood systems by Rindler et al. [32] and Bockel et al. [34] further revealed differences in mechanical responses under wet conditions among the adhesive, the regions adjacent to the interface, and the wood substrate. In contrast, Yuan et al. [115] provided more direct comparative evidence linking interfacial structure to wet shear strength by varying the activation state of the wood surface while maintaining the same adhesive formulation. Together, these studies provide insights into the respective roles of chemical curing, local material properties, and interfacial bonding in joint performance. Nevertheless, no single characterization scale can fully account for the mechanisms governing wet-state failure.

Differences in water exposure protocols further limit the applicability of these structure–property relationships. Wang et al. [141] and Li et al. [109] evaluated lignin-modified adhesives under high-relative-humidity and hot-water treatment conditions, respectively, whereas Yousafzai et al. [137] employed an accelerated hydrothermal protocol involving two boiling-water treatments. Because these tests differ in the mode of moisture exposure, temperature, and treatment history, the resulting bond strength values should not be directly compared to rank the water resistance of different adhesive networks. Accordingly, establishing verifiable structure–property relationships between curing and water resistance requires controlling adhesive formulation, wood substrate, hot-pressing conditions, and water exposure protocols within the same adhesive system. Curing conversion, effective network formation, moisture response, local interfacial mechanical behavior, and wet-state failure characteristics should be analyzed in an integrated manner to better distinguish the contributions of bondline cohesion, interfacial load transfer, and wood substrate response to wet bonding performance.

## 6. Challenges and Future Perspectives

The structural heterogeneity of lignin continues to limit the predictability of its curing reactions. Technical lignins from different sources vary in molecular weight, degree of aromatic condensation, and accessibility of reactive sites, while functional-group content alone does not directly reflect the extent to which these groups participate in crosslinking. Establishing relationships among lignin structural parameters, reactive-site utilization, and curing behavior, together with regulating reactivity through fractionation and selective functionalization, represents an important direction for achieving greater control over curing.

Feedstock purity and batch-to-batch variability remain challenges to reproducible lignin reactions. Clearly defining lignin purity and isolation conditions, together with structural characterization, is essential because similar purity does not ensure equivalent reactivity. Future studies should distinguish variation within the same feedstock batch from differences between batches under consistent formulation and reaction conditions.

The quantitative relationship between curing kinetics and crosslinked-network formation remains insufficiently understood. Existing studies primarily characterize curing processes using apparent activation energy, degree of cure, and gelation time. However, chemical conversion, network percolation, and vitrification exhibit distinct evolution characteristics during curing. Combining in situ spectroscopy, dynamic rheology, and thermomechanical analysis to investigate the evolution of chemical linkages and network structure during curing, while examining the coupled effects of wood moisture content, adhesive penetration, and hot-pressing conditions, would help establish relationships between curing behavior and bond strength development in actual wood joints.

The moisture stability of crosslinked networks and its contribution to wet bonding performance require further investigation. The chemical stability of network linkages, residual hydrophilic groups, and structural homogeneity jointly influence water uptake, swelling, and the mechanical response of the adhesive layer. Correlating the effective degree of crosslinking with moisture uptake and diffusion, wet-state viscoelasticity, and strength retention could help clarify the effects of different network structures on water resistance and establish quantitative structure–property relationships between cured network structures and wet bonding performance.

Elucidating the mechanisms of wet-state failure at the wood–adhesive interface remains another pressing challenge. Moisture-induced swelling of wood, plasticization of the adhesive layer, and changes in interfacial stresses may jointly influence the failure behavior of bonded joints. Integrating local deformation measurements, fracture morphology analysis, and wetting–drying cycle tests would help distinguish cohesive failure within the adhesive layer, interfacial debonding, and wood substrate failure. Harmonizing curing conditions and water-exposure protocols would further support the evaluation and prediction of the long-term service performance of lignin-based wood adhesives.

## 7. Conclusions

This review systematically examines the molecular structure and reactivity, curing reaction mechanisms, crosslinked network formation, and water resistance of lignin-based wood adhesives, with particular emphasis on the relationships between curing behavior and wet bonding performance. Existing studies indicate that lignin molecular weight, the degree of aromatic condensation, and reactive-site accessibility collectively influence its curing reactivity. Phenolation, demethylation, epoxidation, and fractionation can modify the reactivity of lignin; however, their effectiveness depends on the utilization of reactive sites and their compatibility with the curing system.

Different lignin-based adhesives form crosslinked networks through condensation, epoxy ring-opening, and addition reactions involving hydroxyl groups. Curing kinetics studies have revealed the evolution of reaction rates, gelation, and vitrification, although the extent of chemical conversion does not necessarily correspond directly to effective network formation. During wood bonding, adhesive flow, wetting, penetration into wood pores, and the moisture condition of the wood further influence bondline structure and bond strength development.

The water resistance of lignin-based adhesives is jointly governed by the chemical stability of the cured network, residual hydrophilic groups, network spatial architecture, and the wood–adhesive interface. Effective crosslinking can restrict network swelling and improve wet bonding performance in certain systems, but increased curing reactivity does not necessarily lead to a corresponding improvement in water resistance. Moisture-induced plasticization of the adhesive layer, swelling of wood, and changes in interfacial stresses further affect joint load-bearing capacity and failure modes. Therefore, the relationship between curing behavior and water resistance in lignin-based wood adhesives is inherently multiscale, involving the combined effects of molecular structure, crosslinked networks, and bonding interfaces. Understanding the interactions among these structural factors is essential for simultaneously optimizing curing efficiency and wet bonding stability.

## Figures and Tables

**Table 1:** Modification and fractionation strategies for lignin and their applications in adhesive systems.

Strategy	Lignin Feedstock	Treatment and Subsequent Application	Main Finding	Ref.
Phenolation	Wheat straw lignin	Acid-catalyzed phenolation	Phenol incorporation and changes in interunit linkages	[46]
Demethylation	Kraft lignin	Sulfobutylation followed by demethylation; PF resins	Combined solubility adjustment and functional-group conversion	[48]
Hydroxymethylation	Kraft lignin	Co-hydroxymethylation with phenol	Methylene linkages mainly between hydroxymethylated phenol units under tested conditions	[49]
Glyoxal modification	Lignin models and resins	Reaction with glyoxal	NMR evidence for glyoxal-derived linkages in cured resin	[50]
Direct hydroxyl utilization	Fractionated hardwood kraft lignin	Crosslinking with epichlorohydrin	Hydroxyl participation and new oxygen-containing linkages	[51]
Hydroxypropylation	Kraft lignin	Hydroxypropylation; PU adhesives	Treatment-dependent thermomechanical properties and bond strength	[52]
Reductive amination	Enzymatic hydrolysis lignin	Fenton oxidation and reductive amination; ESO/SPI adhesives	Improved wet bonding relative to the SPI control	[53]
Amination	Fractionated lignin	Reaction with bis(six-membered cyclic carbonate); NIPU adhesives	Bonding demonstrated on wood and other substrates	[54]
Amination	Lignin	Activated tannin/MCC-reinforced NIPU	At 30% MCC: curing at 130 °C; dry/wet strength: 1.89/0.85 MPa	[55]
Amino functionalization	Lignin	KH550 modification; crosslinking with ENR	Dry/wet strength: 1.91/1.24 MPa; wet test: 63 °C, 3 h	[56]
Fractionation–phenolation	Hardwood alkali lignin	Fractionation and phenolation; LPF resins	Greater covalent incorporation of lower-MW, less-condensed fractions	[47]
Fractionation–epoxidation	Enzymatic hydrolysis lignin	Ethanol fractionation and epoxidation; hybrid NIPU	Reactive incorporation into a hybrid polymer network	[57]

**Table 2:** Effects of lignin characteristics and formulation variables on reported curing kinetics.

Study	Resin System and Comparison	Analysis	Main Finding
Tejado et al. [20]	Novolac resins with 25% and 45% lignin substitution	DSC; KAS analysis	Kinetic changes associated with reactive structures and viscosity evolution
Song et al. [22]	PF, LPF, and DLPF	Kissinger analysis; gelation measurement	Apparent activation energies: 77.7, 90.2, and 85.3 kJ/mol, respectively; shorter gelation time for DLPF
Ferdosian et al. [73]	Epoxies from depolymerized organosolv or kraft lignin; DDM or DETA curing	Non-isothermal DSC	Lignin source and curing agent affected the conversion dependence of activation energy
Ferdosian et al. [74]	Lignin-based epoxy components blended with bisphenol A-based epoxy	Curing analysis	Early-stage acceleration at 25 wt%; later-stage inhibition above 50 wt%

**Table 3:** Effects of network design and formulation on the water resistance of selected lignin-based adhesives.

System and Reference	Network Design or Formulation Variable	Water-Related Evaluation	Main Reported Outcome
HBPA-functionalized lignin/epoxy [101]	HBPA-to-epoxy ratio and reactive-component loading	Mass retention after 63 °C water exposure for 6 h; water swelling	Mass retention: 35.5% for uncrosslinked oxidized lignin versus 67.9–71.9% for crosslinked formulations. At an HBPA/epoxy mass ratio of approximately 0.6, swelling decreased from 90.3% to 75.7%; an amino-rich formulation showed 106.2% swelling.
Polyacrylamide-grafted lignin/epoxy [104]	Grafted chain segments combined with epoxy crosslinking	Moisture uptake; mass retention; wet shear strength after 63 °C water exposure for 3 h	Relative to unmodified lignin adhesive, moisture uptake decreased by 83.2% and mass retention increased by 331.5%; wet shear strength reached 1.26 MPa.
Soy protein/lignin/PAE [105]	Lignin and PAE addition levels	Wet bond strength; adhesive spreadability	Addition of 1% lignin and 1% PAE increased wet strength from 1.8 to 3.8 MPa. Higher additions reduced spreadability without further improving bonding performance.

## Data Availability

No new data were created or analyzed in this study. Data sharing is not applicable to this article.

## Abbreviations

The following abbreviations are used in this manuscript:

1C-PUR	One-component polyurethane
ABES	Automated Bonding Evaluation System
AFM	Atomic force microscopy
ALCNFs	Aldehyde-functionalized lignin-containing cellulose nanofibrils
APTES	3-Aminopropyltriethoxysilane
APVA	Aminated poly(vinyl alcohol)
ASTM	ASTM International
CLSM	Confocal laser scanning microscopy
CLT	Cross-laminated timber
CML	Carboxymethylated lignin
CT	Computed tomography
DBU	1,8-Diazabicyclo[5.4.0]undec-7-ene
DCB	Double cantilever beam
DDM	4,4′-Diaminodiphenylmethane
DETA	Diethylenetriamine
DIC	Digital image correlation
DL	Dioxane-extracted lignin
DLPF	Demethylated lignin–phenol–formaldehyde
DMA	Dynamic mechanical analysis
DSC	Differential scanning calorimetry
EHL–ESO	Enzymatic hydrolysis lignin–epoxidized soybean oil
EIS	Electrochemical impedance spectroscopy
EN	European Standard
ENR	Epoxidized natural rubber
EPI	Emulsion polymer isocyanate
EPL	Epoxidized lignin
FEA	Finite element analysis
FPL	Formaldehyde-protected lignin
FRP	Fiber-reinforced polymer
GPC	Gel permeation chromatography
H	p-Hydroxyphenyl (lignin structural unit)
HBPA	Hyperbranched polyamide
HMF	5-Hydroxymethylfurfural
HMTA	Hexamethylenetetramine
HSQC	Heteronuclear single quantum coherence
ISO	International Organization for Standardization
KAS	Kissinger–Akahira–Sunose
LCL	Cysteine-functionalized lignin
LCNFs	Lignin-containing cellulose nanofibrils
LPF	Lignin–phenol–formaldehyde
LPU	Lignin-based polyurea
LVL	Laminated veneer lumber
MCC	Microcrystalline cellulose
MDI	4,4′-Diphenylmethane diisocyanate
MEA	Monoethanolamine
MUF	Melamine–urea–formaldehyde
NIPU	Non-isocyanate polyurethane
NMR	Nuclear magnetic resonance
PAE	Polyamidoamine–epichlorohydrin
PEG	Polyethylene glycol
PF	Phenol–formaldehyde
PLEP	Phenolated lignin epoxy resin
pMDI	Polymeric methylene diphenyl diisocyanate
PRF	Phenol–resorcinol–formaldehyde
PUR	Polyurethane
PVAc	Poly(vinyl acetate)
RF	Resorcinol–formaldehyde
SEC	Size-exclusion chromatography
SEM–EDS	Scanning electron microscopy–energy-dispersive X-ray spectroscopy
SP	Soy protein
SPI	Soy protein isolate
TTT	Time–temperature–transformation
UF	Urea–formaldehyde
